# Mechanical compression induces neuronal apoptosis, reduces synaptic activity, and promotes glial neuroinflammation in mice and humans

**DOI:** 10.1073/pnas.2513172122

**Published:** 2026-01-02

**Authors:** Maksym Zarodniuk, Anna Wenninger, Julian Najera, Jihaeng Lee, Jack Markillie, Cameron MacKenzie, Jenny Bergqvist-Patzke, Bianca Batista, Megna Panchbhavi, R’nld Rumbach, Alice Burchett, Charles Sander, Meenal Datta, Christopher Patzke

**Affiliations:** ^a^Department of Aerospace and Mechanical Engineering, University of Notre Dame, Notre Dame, IN 46556; ^b^Department of Biological Sciences, University of Notre Dame, Notre Dame, IN 46556; ^c^Department of Chemistry and Biochemistry, University of Notre Dame, Notre Dame, IN 46556; ^d^Department of Chemical and Biomolecular Engineering, University of Notre Dame, Notre Dame, IN 46556; ^e^Department of Applied and Computational Mathematics and Statistics, University of Notre Dame, Notre Dame, IN 46556

**Keywords:** solid stress, glioblastoma, hypoxia, gliosis

## Abstract

Mass effect, characterized by the compression and deformation of neural tissue from space-occupying lesions, can lead to debilitating neurological symptoms and poses a significant clinical challenge. In the primary brain tumor glioblastoma (GBM), we have shown previously that compressive solid stress originating from the growing tumor reduces cerebral blood flow, leading to neuronal loss, increased functional impairment, and poor clinical outcomes. However, the direct effects of compression on neurons and the underlying biophysical mechanisms are poorly understood. Here, using multiscale compression systems and physiologically relevant in vitro and in vivo models, we find that chronic mechanical compression induces neuronal apoptosis and loss of synaptic puncta, leading to disrupted neural network activity, as assessed by calcium imaging. This is accompanied by increased HIF-1 signaling and upregulation of downstream stress-adaptive genes in neurons. We further show that chronic compression triggers AP-1–driven gene expression in glial cells, promoting a neuroinflammatory response. Together, these findings reveal that solid stress directly contributes to neuronal dysfunction and inflammation caused by GBM by activating distinct pathways that can be targeted in future studies for neuroprotection.

## Significance

Glioblastoma, the deadliest primary brain tumor in adults, exerts physical forces on surrounding brain tissue, leading to neuronal damage. In the present study, by applying multiple model systems, we show that chronic mechanical compression triggers neuronal apoptosis, disrupts synaptic communication between neurons, and reduces neural network activity. We also find that compression activates inflammatory pathways in both neurons and glia, with reactive astrocytes contributing to the neuroinflammatory environment that accompanies neuronal loss. These findings reveal how compression exerted by space-occupying lesions may contribute to patients’ cognitive and motor impairments. This work lays the groundwork for therapies that protect neurons from mechanical injury, with relevance not only to glioblastoma but also to other neurological diseases that present with mass effect.

Several central nervous system pathologies such as traumatic brain injury (TBI), multiple sclerosis (MS), benign and malignant brain tumors are known to present with mass effect, where a focal lesion or contusion compresses and deforms surrounding neural tissue due to the space occupied by increased cell numbers (arriving via circulation and/or proliferating locally) and/or extracellular matrix (ECM) deposition within a confined area. Such deformations, including ventricular compression and displacement of the brain midline, are immediately visible on MRI. Clinicians frequently observe both fluid (i.e., edema, resulting from disrupted or leaky vasculature and/or cerebrospinal fluid accumulation) and solid components of these lesions and evaluate these criteria during diagnosis (1–3). Intracranial mass effect (as opposed to compression of the spinal cord or peripheral nervous system) is particularly unique and insidious, as the rigid skull confines and compounds these mechanical forces (4). As a result, patients often present with debilitating neurological symptoms including sensory, motor, cognitive, and behavioral dysfunction (5, 6).

In the case of glioblastoma (GBM), compressive solid stresses are generated by elastic components of the tumor microenvironment, namely the cells and ECM (7–9). Additionally, peritumoral edema promotes increased intracranial pressure and further deforms brain tissue (10). Similar space-occupying lesions, presenting with an overlapping but distinct set of symptoms, have been observed in other neurological disorders such as tumefactive MS (11, 12) and severe TBI (13–15). Both edema and solid stress contribute to brain deformation and have been measured/estimated and investigated as prognostic/predictive biomarkers in brain tumor and TBI (2, 4, 16, 17). In GBM, the extent of peritumoral edema is an independent prognostic factor; however, its direct effect on neurological performance is unclear (4, 18). In contrast, solid stress has been linked to increased functional impairment, reduced cerebral blood flow (CBF), and poorer clinical outcomes in GBM patients (4, 19). Our prior preclinical investigations have shown that chronic brain compression using mechanical compression techniques in vivo lead to similar effects, such as reduced vessel perfusion, neuronal loss, and neurological dysfunction, even in the absence of a tumor (4).

Growing brain masses, such as tumors, cause tissue compaction that generates both compressive and tensile forces. These mechanical stresses can compress, shear, and stretch axons, triggering a series of cellular processes that ultimately contribute to neuronal dysfunction and death (20). In TBI, acute mechanical damage has been shown to trigger long-term dysregulation of intracellular calcium metabolism (21, 22), excitotoxicity (23), Wallerian degeneration (24), and neuroinflammation (25, 26) in injured neurons. In addition, mechanical trauma can lead to disinhibited astrocytic and microglial activation, resulting in local neuroinflammation, reactive gliosis, and cytotoxic edema, contributing to secondary injury (20). While these mechanisms are well documented in the context of acute injury, the effects of chronic compressive forces, such as those produced by a growing tumor, remain less understood.

In this study, we examine how chronic mechanical compression directly affects neurons. To this end, we use engineered systems both in vitro and in vivo that simulate the compressive forces experienced by the neural tissue directly adjacent to the GBM tumor. We have previously measured these forces in mouse models of GBM (4, 27) and more recently in human subjects (28). Given that GBM grows primarily in the cortical regions where excitatory neurons comprise approximately 80% of the neuronal population (29), our in vitro model combines induced pluripotent stem cell (iPSC)-derived excitatory neurons and glia to represent the predominant cell populations affected by compressive stresses. Here, we show that chronic compression of these cells leads to neuronal and synaptic puncta loss, as well as impaired neural network activity. Additionally, we find that compression activates reactive gene expression programs in astrocytes and microglia, including upregulation of neuroinflammatory cytokines. Our findings provide insights into how solid stress contributes to neuroinflammation and neurodegeneration in GBM and other neurological diseases and inform future strategies to reduce these deficits in patients suffering from mass effect.

## Results

### Compression Induces Neuronal Loss In Vitro.

**Fig. 1.**
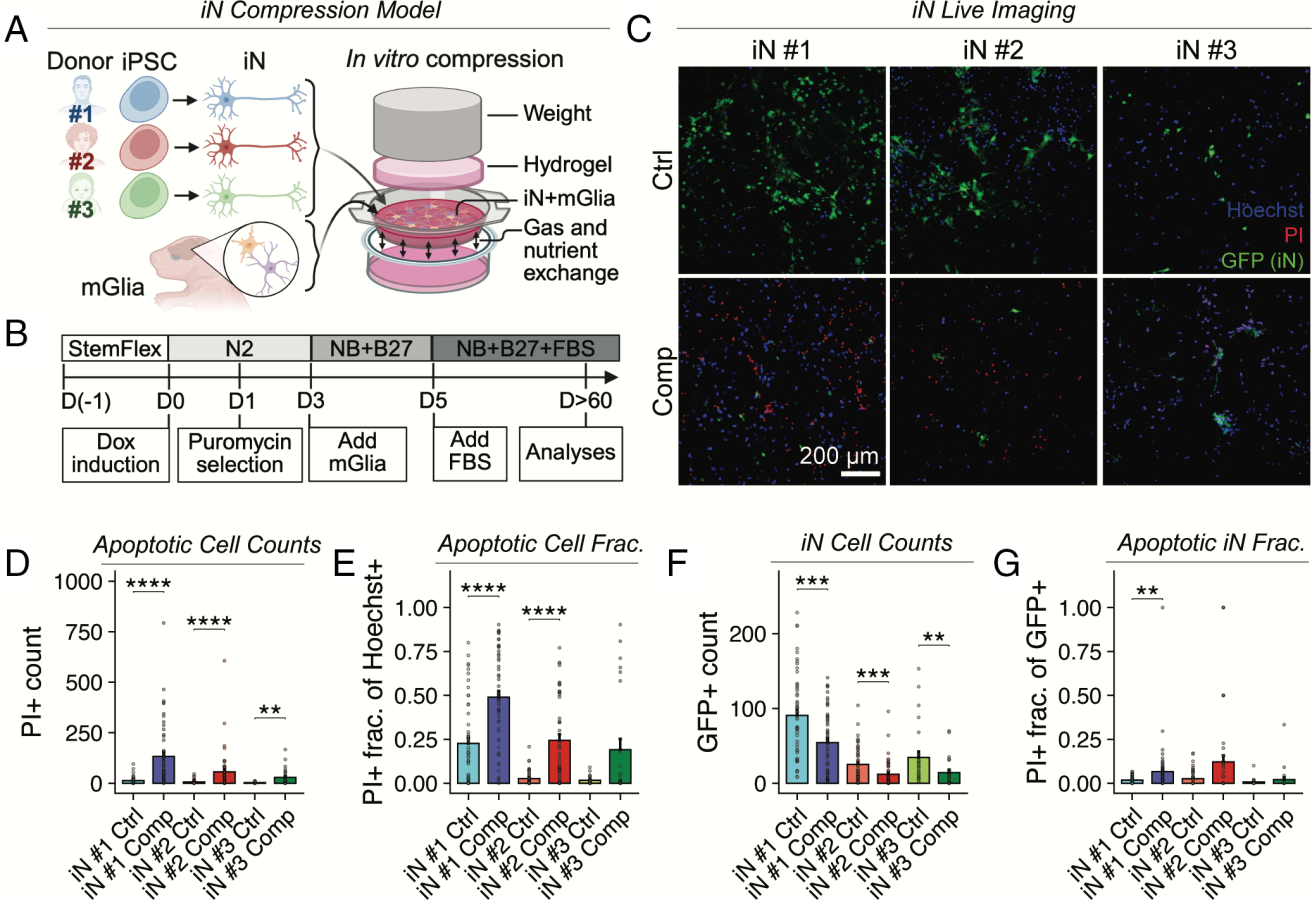
Compressive solid stress induces neuronal cell death in human Ngn2-induced neurons. (*A*) Schematic of the in vitro compression system used to apply 0.14 kPa of compressive stress on human induced excitatory neurons (iNs), mimicking solid stress exerted by a GBM tumor. iNs are derived from human iPSCs from diverse donors and are cocultured with mGlia to facilitate neuron maturation and synapse formation. (*B*) Diagram showing the protocol used for iN induction from iPSCs. (*C*) Representative images of iN cultures stained with PI and Hoechst 33342 after 24-h compression. GFP expression in iNs is largely restricted to the nucleus. (*D*) Apoptotic cell numbers are quantified as the number of PI-positive cells per field of view. (*E*) Apoptotic cell fraction is defined as the proportion of Hoechst 33342-positive cells overlapping with PI+ cells in each field of view. (*F*) Total iN cell counts correspond to the number of GFP+ cells per field of view. (*G*) Apoptotic iN fraction is defined as the proportion of GFP+ cells overlapping with PI+ cells per field of view. Mann–Whitney *U* test with Holm–Bonferroni adjustment for multiple comparisons using n = 4 biological replicates for iN #3, n = 7 biological replicates in iN #2 and iN #1 neurons per group; the data are based on 7 independent experiments; Error bars: mean ± SEM. Statistical significance is indicated as follows: ns = *P* ≥ 0.05; *P < 0.05; **P < 0.01; ***P < 0.001; ****P < 0.0001.

Solid stress is known to impair neurological function in vivo; however, its specific molecular effects on neurons are poorly understood. To investigate this, we used an in vitro compression system to apply controlled mechanical compression to human neurons cocultured with murine astrocytes (Fig. 1*A*). Using our established protocol (30), we generated induced excitatory neurons (iNs) by forced expression of the transcription factor neurogenin 2 (Ngn2) in human iPSCs from three donors of varying sexes and ethnic origins (referred to as iN #1-3) (Fig. 1*B*). We applied 0.14 kPa of compressive stress, mimicking forces exerted by a growing GBM tumor as measured in our prior in vivo studies (4) and GBM patients (28). After 24 h of compression, the iN cultures were stained with propidium iodide (PI) and Hoechst. Live imaging (Fig. 1*C*) revealed a more than twofold increase in the number and frequency of PI+ cells in compressed iNs compared to controls (Fig. 1 *D* and *E*), indicating loss of membrane integrity consistent with late apoptosis or necrosis. To specifically assess neuronal viability, we used GFP-expressing iNs to distinguish them from glia. Compressed iN cultures from iPSCs #1 and #2 showed significant neuronal loss (Fig. 1*F*) and increased numbers of PI+ neurons (Fig. 1*G*). We also observed an increase in GFP-PI+ cells, which may represent either glial cells undergoing cell death or dead neurons that lost GFP expression (*SI Appendix*, Fig. S1*A*). Nonetheless, we found a slight increase in the overall number of cells (*SI Appendix*, Fig. S1*B*), as measured by Hoechst staining, indicating heightened proliferation of glial cells under compression. These results demonstrate that pathophysiological levels of mechanical compression directly lead to neuronal cell death. Among the three iPSC lines, iN #1 neurons consistently exhibited the most robust and reproducible response to compression and thus were selected for further functional and morphological analyses.

### Solid Stress Reduces the Number of Synaptic Puncta and Neuronal Network Activity.

**Fig. 2.**
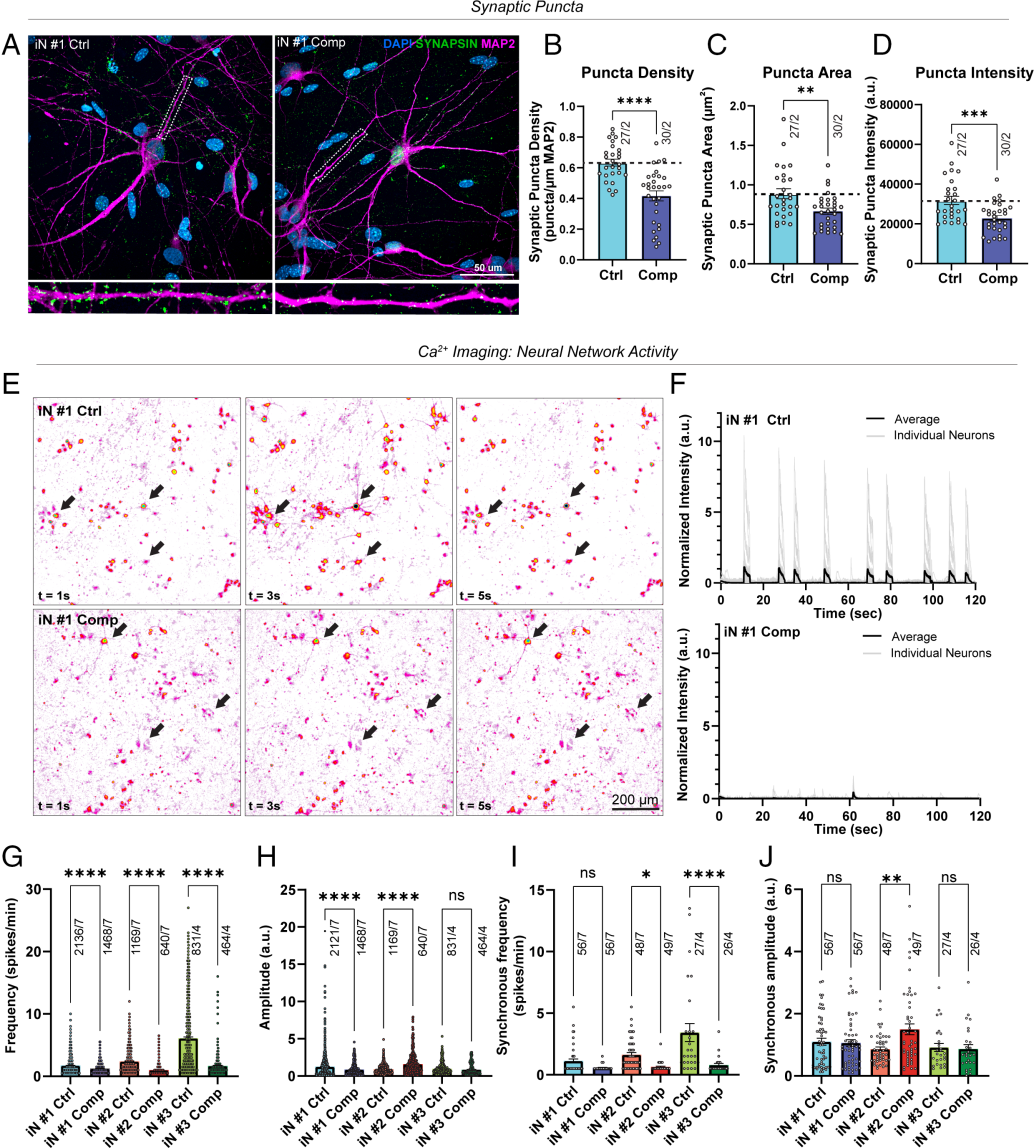
Compression decreases the number of synaptic puncta in human neurons. (*A*) Representative confocal images of iN #1 compressed and uncompressed human neurons, showing decreased levels of synaptic puncta. (blue = DAPI, green= Synapsin, magenta = MAP2). (Scale bar, 50 μm.) (*B*–*D*) Quantification of synaptic puncta density (*P* = <0.0001), area (*P* = 0.0036), and intensity (*P* = 0.0005) in iN #1. Student’s unpaired *t*test n = 27 uncompressed images, n = 30 compressed images, from 2 biological replicates for each condition. (*E*) Representative heat maps for calcium imaging at 2 s intervals. (Scale bar, 200 μm.) (*F*) Representative traces of GCamp8 transients showing fluorescence intensity changes (ΔF/F0) over time. Compressed neurons show decreased event frequency. (*G*–*J*) Quantification of calcium imaging of iN #1-3 compressed and uncompressed human neurons. Frequency (iN #1 *P* = <0.0001, iN #2 *P* = <0.0001, iN #3 *P* = <0.0001) and amplitude plots (iN #1 *P* = <0.0001, iN #2 *P* = <0.0001, iN #3 *P* = 0.2411) show the activity of individual neurons, while synchronous frequency (iN #1 *P* = 0.3312, iN #2 *P* = 0.0121, iN #3 *P* = <0.0001) and amplitude (iN #1 *P* = 0.9998, iN #2 *P* = 0.0032, iN #3 *P* = >0.9999) show the network activity. One-way ANOVA, n = 4 biological replicates in iN #3, n = 7 biological replicates in iN #2 and iN #1 per group. Error bars: mean ± SEM.

Given the pronounced neuronal loss observed under compressive stress, we next investigated whether mechanical compression also compromises synaptic integrity and neuronal network activity. Labeling for synapsin, a presynaptic vesicle marker, and MAP2, a marker for postsynaptic dendrites (together serving as a proxy for synapses), we analyzed synaptic morphological changes based on three complementary metrics: synaptic puncta density, puncta area, and puncta intensity, which reflect putative synapse number and clustering (Fig. 2 *A*–*D*). Upon compression, we detected a decrease of all three parameters, indicating a reduction of the number and clustering of synaptic connections. Additionally, we analyzed the morphology of human neurons with and without compression. Compressed neurons from iPSCs #1 (iN #1) exhibited modestly reduced dendritic arbor complexity, inverse to the normal trajectory of neuronal development (*SI Appendix*, Fig. S2). To probe for the functional consequences following mechanical compression, we performed GCaMP8m-based calcium imaging (Fig. 2 *E* and *F*). In compressed iNs, we detected a robust decrease in the single-neuron event frequency (Fig. 2 *G* and *H*) and a less pronounced decrease in the synchronous event frequency (Fig. 2 *I* and *J* and Movie S1). These results are indicative of reduced neuronal network activity caused by neuronal and synaptic puncta loss.

### Solid Stress Activates Hypoxia Response, Proinflammatory, and Apoptosis Pathways in Human Neurons and Murine Glia (mGlia).

**Fig. 3.**
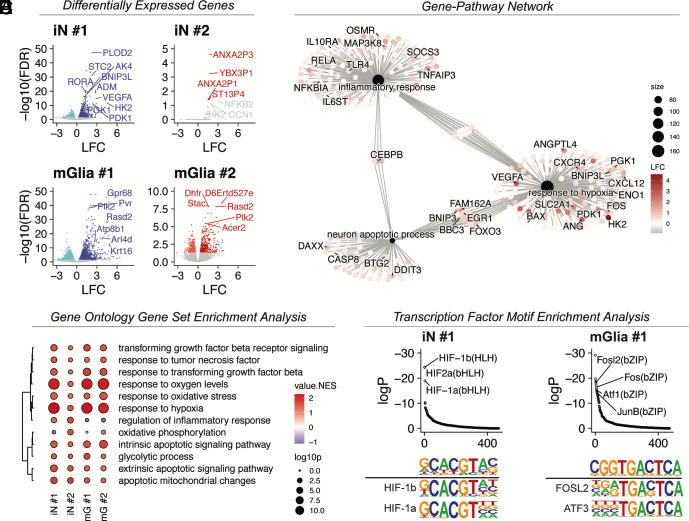
Compression activates hypoxia, apoptosis, and inflammatory pathways in human neurons. Volcano plots for human neurons (iNs) (*A*) and mGlia (*B*) showing up and downregulated genes in dark and light hues, respectively. Top differentially expressed genes are labeled and are enriched in hypoxia response genes; LFC: log fold change. (*C*) Dot plot showing NES for iNs and mGlia and select GO terms. Clustering is based on semantic similarity of GO terms. NES: normalized enrichment score; log10P: negative base 10 logarithm of the adjusted *P* value. (*D*) GO term to gene graph showing relative expression of leading edge genes for iN #1 in three select pathways. size: number of genes in the leading edge. (*E*) Transcription factor motif enrichment analysis of upregulated (FDR < 0.05, LFC > 0) genes in iN #1 and mGlia. Motifs are ranked by log*P* (logarithm of adjusted *P* value) based on cumulative binomial test. At the bottom, sequence logos for enriched motifs are aligned against known transcription factor motifs for comparison. n = 4 biological replicates from 4 experiments for iNs #1 and #2.

To investigate mechanisms by which solid stress contributes to neuronal dysfunction, we performed RNA-seq on iN/mGlia cocultures after 24 h of mechanical compression. To distinguish between neuronal and glial transcriptional responses, sequencing reads were aligned separately to the human and mouse reference genomes using BBmap (Fig. 3 *A* and *B* and *SI Appendix*, Fig. S1*C*). In human iNs, gene set enrichment analysis (GSEA) identified hypoxia response as the most strongly activated pathway (Fig. 3*C*). Leading edge analysis highlighted increased expression of genes involved in glycolysis (*HK2, PDK1, PGK1, ENO1, SLC2A1*), angiogenesis (*VEGFA, ANG, ANGPTL4, CXCL12, CXCR4*), and apoptosis (*FAM162A, BNIP3, BNIP3L, BBC3, CASP8, BAX*), indicating a mix of prosurvival and proapoptotic hypoxia-driven responses (Fig. 3*D*). Additionally, we observed significant activation of the neuronal apoptotic program that included both overlapping and distinct markers from the hypoxia response, such as *DAXX, FOXO3, DDIT3 (*CHOP*), PML, ATM, CHEK2, E2F1,* and *PARP3* (Fig. 3*D*). Notably, we detected activation of inflammatory signaling pathways, including the NF-κB, AP-1, and JAK/STAT pathways, as evidenced by elevated expression of *RELA, NFKBIA, TNFAIP3, TLR4, CEBPB, MAP3K8, FOS, JUN, SOCS3, IL6ST, IL10RA,* and *OSMR* (Fig. 3*D*). These findings suggest that neuronal responses to mechanical stress may be compounded by inflammatory signals released from neighboring glia.

In mGlia, mechanical compression similarly activated hypoxia and intrinsic apoptotic pathways (Fig. 3*C*). In addition, mGlia exhibited strong upregulation of NF-κB pathway transcription factors (*Fosl1, Fosl2, Jun, Junb, Fosb, Fos, Rel*) and proinflammatory cytokines (*Cxcl1, Cxcl2, Cxcl3, Il23a*), consistent with astrocytic and/or microglial activation (astro-/microgliosis) (Dataset S1). To further define the glial cell types contributing to this response, we performed cell type deconvolution using the Tabula Muris single-cell RNA-seq atlas (31). This analysis identified astrocytes as the top-scoring cell type (*SI Appendix*, Fig. S1*D*).

To determine whether gene expression changes are consistent across iPSC donors #1 and #2, we performed a rank–rank hypergeometric overlap (RRHO) analysis using genes ranked by log_2_ fold change (*SI Appendix*, Fig. S3*A*). The analysis revealed significant hypergeometric p-values in the top right and bottom left quadrants, indicating highly concordant up- and down-regulated genes between the two datasets (*SI Appendix*, Fig. S3*B*). Moreover, we found significant correlation between expression fold changes in neurons derived from donors #1 and #2 and glia, with numerous genes exhibiting concordant upregulation, including *VEGFA* and *HK2*—two canonical Hypoxia-Inducible Factor 1 (HIF1)-inducible targets (*SI Appendix*, Fig. S3*C*). Finally, pathway-level GSEA demonstrated upregulation of hypoxia, apoptosis, and inflammatory response pathways in iNs #1 and #2, while NF-κB signaling was consistently upregulated in respective murine glial cultures (*SI Appendix*, Fig. S3*D*). These results indicate that solid stress responses are conserved across experimental models.

To identify putative upstream regulators driving these responses, we conducted transcription factor motif enrichment analysis. In neurons, HIF-1 and HIF-2 binding motifs were strongly enriched in transcription start sites (TSS) of upregulated genes, consistent with elevated hypoxia signaling (Fig. 3*E*). In glia, Activator-Protein 1 (AP-1) transcription factor motifs were significantly enriched, suggesting that the glial inflammatory response may be driven in part by AP-1 signaling. Together, these data suggest that mechanical compression activates HIF-1, inflammatory, and apoptotic signaling in both neurons and glia.

### Solid Stress Activates Reactive Glial Gene Expression Programs in Human Astrocytes and Microglia.

**Fig. 4.**
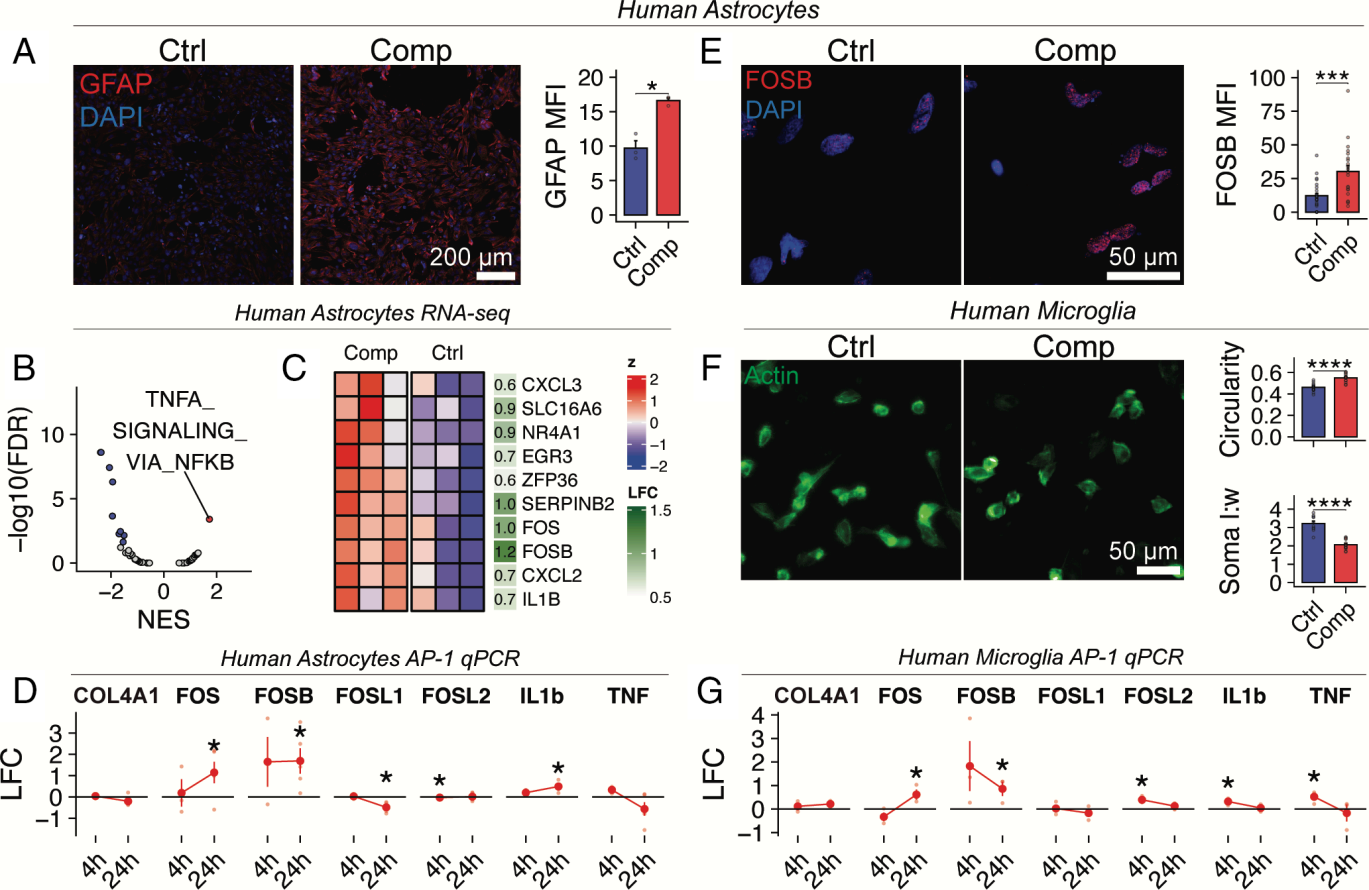
Mechanical compression activates astro- and microgliosis via upregulation of the AP-1 signaling pathway. (*A*) Immunofluorescence analysis of GFAP, a marker of reactive astrocytes, in compressed immortalized human astrocytes. (n = 3 biological replicates per group from one experiment). (*B*) Volcano plot showing top dysregulated pathways in astrocytes based on GSEA of MSigDB HALLMARK gene sets; FDR: false discovery rate; NES: normalized enrichment score. (*C*) Normalized and scaled expression of top differentially expressed genes in astrocytes from the HALLMARK_TNFA_SIGNALING_VIA_NFKB gene set. LFC: log fold change. (*D*) qPCR gene expression quantification of NF-κB and AP-1 pathway genes in compressed astrocytes; n = 3 biological replicates for the 4 h time point and n = 5 biological replicates for the 24 h time point. (*E*) Immunofluorescence analysis of FOSB in compressed astrocytes (n = 4 biological replicates per group from one experiment). (*F*) Changes in morphology of C20 immortalized human microglia are quantified as circularity and soma length:width ratio using ImageJ based on actin staining (n = 3 biological replicates per group from one experiment). (*G*) qPCR gene expression quantification of NF-κB and AP-1 pathway genes in compressed microglia (3 biological replicates for all timepoints). Error bars, mean ± SEM. All *P* values are based on Student’s *t*tests. Stars indicate significance levels: *P < 0.05, **P < 0.01, ***P < 0.001, ****P < 0.0001. For (*D* and *G*) *P* values are calculated by comparing comp. v.s. ctrl at each timepoint. LFC is calculated using the ddCt method; dCt is calculated relative to GAPDH expression.

Astrocytes can become reactive in response to both direct mechanical injury and signals released by stressed or dying neurons (32). To determine whether solid stress can directly trigger astrogliosis in the absence of neurons, we subjected immortalized human astrocytes (IHAs) to in vitro compression. After 24 h, compressed astrocytes showed increased expression of GFAP—a canonical marker of reactive astrocytes (Fig. 4*A*).

To explore the mechanisms driving this response, we performed RNA-seq on compressed IHAs. GSEA revealed significant activation of the NF-κB signaling pathway (Fig. 4 *B* and *C*), including immediate-early AP-1 transcription factors *JUN*, *FOS*, and *FOSB*, similar to the response observed in mGlia. To assess the dynamics of this response, we performed qPCR at 4 and 24 h postcompression. *FOS* and *FOSB* levels increased rapidly at 4 h and remained elevated at 24 h (Fig. 4*D*), indicating sustained activation rather than a transient, acute response. This was further confirmed by immunocytochemistry, which showed increased nuclear translocation of Fos-B under compression (Fig. 4*E*). IL1B was specifically induced at 24 h, further supporting a chronic inflammatory response. Notably, TNF-α—a key upstream activator of canonical NF-κB signaling—was not upregulated, pointing instead to a noncanonical, potentially mechanosensitive mode of NF-κB activation.

In vivo, astrogliosis may also result from indirect effects of solid stress, such as hypoxia resulting from vascular compression, as we have shown previously (4). To determine whether changes in local oxygen tension contributed to astrocyte activation, we repeated the compression experiment under hypoxic conditions (1% O_2_) and found that compression still induced upregulation of GFAP (*SI Appendix*, Fig. S4 *A* and *B*). To further rule out the effects of residual hypoxia that may persist even in 1% O_2_, we used the HypoxiTRAK™ oxygen-sensing probe, which showed no increase in biologically relevant hypoxia levels under compression (*SI Appendix*, Fig. S4 *C* and *D*). Consistently, HIF1A expression remained unchanged (*SI Appendix*, Fig. S4 *E* and *F*), supporting the conclusion that compression-induced astrogliosis occurs independently of hypoxia.

Because astrocyte reactivity often occurs in concert with microglial activation, we next asked whether microglia also respond directly to compressive stress. Microglia are highly sensitive to tissue damage and can initiate neuroinflammatory cascades that influence both astrocyte reactivity and neuronal survival (33). To assess their response, we examined the effects of compression on the morphology of immortalized human C20 microglia (34–36), which changes rapidly in response to injury (37). Morphological analysis revealed that compressed C20 microglia adopted a more reactive, amoeboid phenotype, with increased circularity and reduced soma length-to-width ratio (Fig. 4*F*) (37). Similar to astrocytes, compressed microglia showed sustained upregulation of FOS and FOSB, but their expression of proinflammatory cytokines TNF-α and IL1B was transient, returning to baseline by 24 h (Fig. 4*G*). These results indicate that mechanical compression directly induces reactive transcriptional programs in both astrocytes and microglia via AP-1 and NF-κB signaling. This glial activation may amplify neuroinflammation and contribute to neuronal injury and death under solid stress.

### Solid Stress Upregulates Hypoxic and Inflammatory Signaling In Vivo in Murine and Human Brains.

**Fig. 5.**
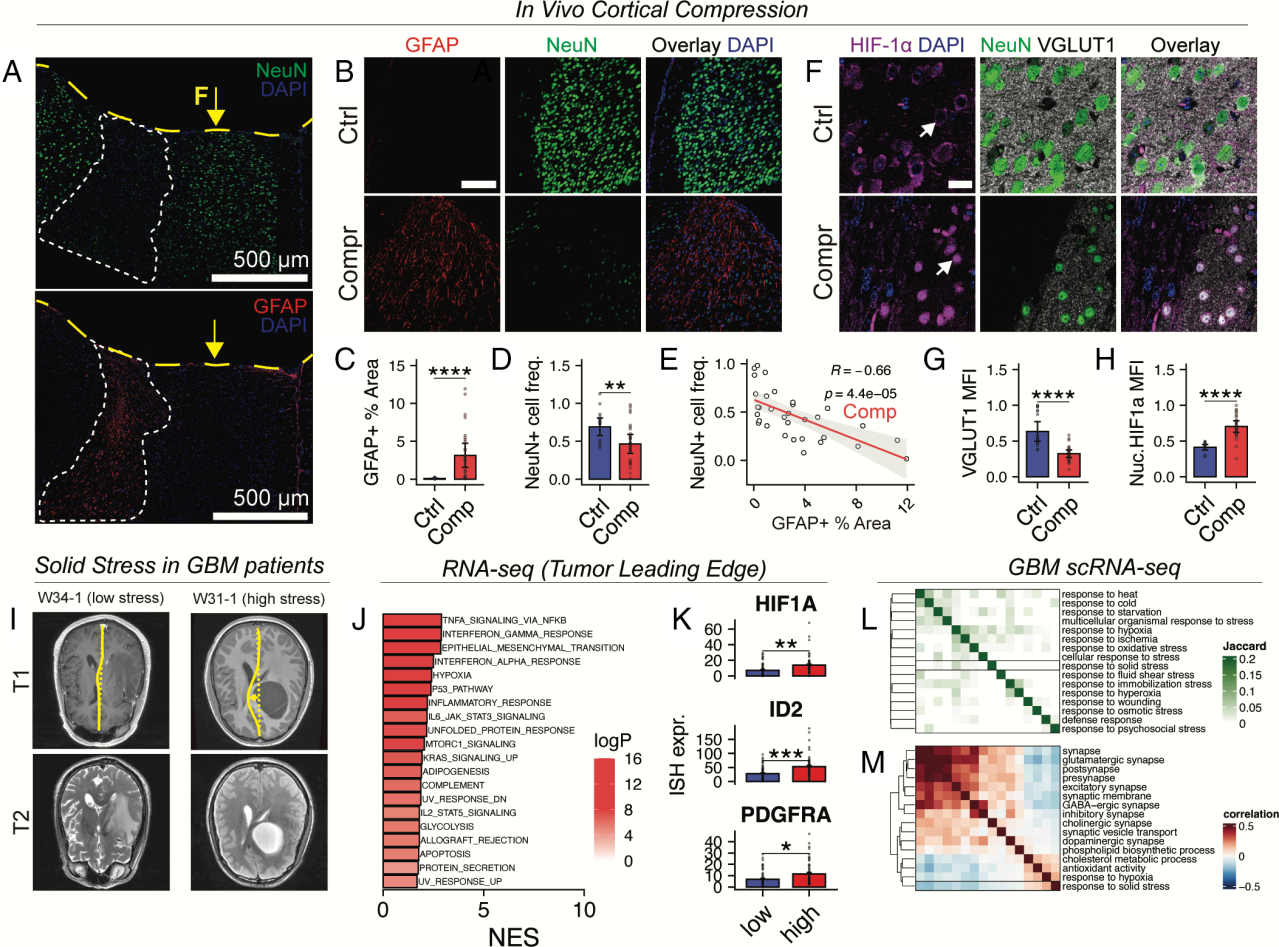
Solid stress promotes hypoxic and inflammatory gene expression programs in vivo. (*A*) Representative immunofluorescence images of cortices subjected to mechanical compression, stained for NeuN (*Top*) or GFAP (*Bottom*). A region lacking NeuN signal, indicative of neuronal loss, and showing increased GFAP staining, consistent with reactive astrogliosis, is outlined with a dashed white line. The area of cortical indentation (compression divot) is marked with a yellow dashed line, and the site of applied compression is indicated by a yellow arrow; F: compressive force. (*B*) Representative images showing NeuN and GFAP expression in the compressed cortex (Scale bar: 100 µm). (*C*–*E*) Quantification of IHC data shown in panel *B*. GFAP % area is quantified as a fraction of pixels above an intensity threshold; neuron frequency (NeuN+/DAPI+) is quantified as the number of NeuN+ nuclei divided by the number of DAPI+ nuclei. (*F*) Representative images showing NeuN, GFAP, and VGLUT1 expression in the compressed cortex (Scale bar: 20 µm). (*G* and *H*) Quantification of IHC data shown in panel *F*. Nuclear HIF-1α mean fluorescence intensity (MFI) is quantified for NeuN+DAPI+ nuclei. VGLUT1 MFI is quantified for the entire tissue region in the field of view. Mann–Whitney *U* test using n = 2 biological replicates for Comp and n = 3 biological replicates for Ctrl from 1 experiment. (*I*) Representative MRI images from GBM patients with high (W31-1) and low (W34-1) estimated solid stress, showing T2-weighted (*Bottom*) and T1 postcontrast (*Top*) scans. (*J*) Top dysregulated pathways identified by GSEA of IvyGAP RNA-seq data from histologically normal “leading edge” regions from patients with high estimated solid stress. NES: normalized enrichment score. logP: negative logarithm of adjusted *P* value. (*K*) Quantification of ISH data in the leading edge region. “Low” and “High” refer to patients with low and high estimated solid stress, respectively. Student’s two-tailed *t* test using n = 9 patients for “high” and n = 10 for “low” groups. (*L*) Jaccard similarity matrix shows overlap between our solid stress response signature and GO gene sets related to other stressors. (*M*) Spearman correlation matrix showing a negative relationship between the solid stress response and synapse maintenance gene expression programs in excitatory neurons from GBM patients. Spearman correlation coefficients were computed separately within each sample, and the resulting per-sample values were averaged to obtain the overall correlation estimate. Stars indicate significance levels: *P < 0.05, **P < 0.01, ***P < 0.001, ****P < 0.0001.

Taken together, the above results demonstrate that mechanical compression directly activates reactive phenotypes in astrocytes and microglia, and induces proapoptotic signaling in neurons. To assess the relevance of these findings in vivo, we applied chronic, localized mechanical compression to the mouse brain using a custom-designed apparatus (*SI Appendix*, Fig. S5*A* and *Materials and Methods*). In compressed cortical regions, we observed regions of dense GFAP+ astroglial scarring (Fig. 5 *A*-*C*), accompanied by loss of NeuN+ neurons (Fig. 5 *B* and *D*). These changes overlapped spatially (Fig. 5*E*), consistent with astrocyte-mediated neurotoxicity. Additionally, astrocytes in the compressed cortical areas had increased nuclear FOSB (*SI Appendix*, Fig. S5 *B* and *C*), providing evidence that the AP-1 signaling pathway may drive the formation of glial scars. The compressed area also showed reduced VGLUT1 (Fig. 5 *F* and *G*), a synaptic marker, concurrent with increased nuclear HIF-1α in viable neurons, indicating rapid synaptic puncta loss (Fig. 5 *F* and *H*).

To determine whether similar patterns occur in human disease, we analyzed data from the Ivy Glioblastoma Atlas Project (38). For each patient, we quantified midline shift (MLS) on T1 postcontrast MRI (Fig. 5*I*), a widely used radiographic marker of mass effect that correlates with poor neurological outcomes in GBM (39, 40) as well as stroke (41–44) and TBI (45–49). To estimate deformations arising due to solid stress, we regressed MLS on edema volume and used the residuals to rank patients based on estimated solid stress (*Materials and Methods* and *SI Appendix*, Fig. S6*A*). Differential expression analysis of RNA-seq data from the tumor leading edge revealed upregulation of NFκB, p53, interferon signaling, and hypoxia response pathways in patients with high estimated solid stress (Fig. 5*J*). In situ hybridization (ISH) data confirmed elevated *HIF1A* expression in the same regions, along with increased levels of oligodendroglial precursor markers *ID2* and *PDGFRA* (Fig. 5*K* an *SI Appendix*, Fig. S6*B*). Gene expression deconvolution using BayesPrism (*SI Appendix*, Fig. S6*C*) revealed increased glycolysis in neurons and elevated NF-κB signaling in astrocytes from patients with high estimated solid stress (*SI Appendix*, Fig. S6*D*), mirroring our experimental findings. While BayesPrism has been less extensively validated for inferring gene expression profiles in nonmalignant cells, the inferred transcriptional changes closely paralleled those observed experimentally in our in vitro and in vivo models.

Finally, to test whether cell-autonomous mechanisms contribute to the synaptic alterations we observed in both in vitro and in vivo models of compression, we analyzed single-nucleus RNA sequencing data (snRNA-seq) from the GBM Cellular Analysis of Resistance and Evolution (CARE) consortium (50, 51). As a proxy for neuronal transcriptional responses to compression, we derived a “solid stress response” gene signature consisting of the top 50 genes upregulated in compressed iN #1 and iN #2 cultures. To ensure that this signature did not reflect generic stress responses (e.g., hypoxia, nutrient deprivation), we computed the Jaccard overlap between our gene set and all relevant child Gene Ontology (GO) terms under the “response to stress” (GO:0006950) root term. The analysis revealed minimal overlap, indicating that our gene signature was largely distinct from other stress-related pathways (Fig. 5*L*). Nonetheless, several genes, including *ADM, HK2, ANG, ANGPTL4, DDIT4, FOSL2, PLOD2, SLC2A1, STC2,* and *VEGFA*, were also shared with GO terms related to hypoxia and oxidative stress responses. Because the expression of these genes can be influenced by multiple stressors, we refined our gene set by excluding any genes present in other stress-associated GO categories, thereby obtaining a solid stress–specific gene signature.

We next used this signature to score excitatory neurons (*SI Appendix*, Fig. S6*E*) and examined its relationship to a manually curated list of gene expression programs associated with synaptic signaling, synapse organization, and neuronal metabolism using Spearman correlation (ρ). In excitatory neurons, the solid stress signature showed a consistent negative correlation with synapse-related and phospholipid biosynthesis gene expression, and a positive correlation with hypoxia response, antioxidant activity, and cholesterol metabolic process (Fig. 5*M*). These observations may be indicative of metabolic and redox adaptation to mechanical stress, in which neurons downregulate synaptic maintenance programs while upregulating pathways that preserve membrane integrity and redox balance. Taken together, these findings suggest that gene expression changes induced by direct chronic compression are associated with transcriptional signatures of synaptic dysfunction in excitatory neurons in GBM patients.

## Discussion

We previously demonstrated that chronic compression of the mouse cortex—at a rate that recapitulates in vivo GBM growth rates—leads to neuronal loss and neurological deficits (4). However, the molecular mechanisms linking compression to these outcomes remained unclear. In this study, we used controlled and mechanistic in vitro and in vivo compression systems that mimic the mechanical forces exerted by GBM. These systems allowed us to examine the direct consequences of chronic mechanical compression on neurons, independent of biochemical remodeling of neurons by GBM cells (52). Our findings demonstrate that compression leads to a marked decrease in the frequency of spontaneous calcium transients in neurons, indicating disrupted intrinsic activity. This loss of network synchrony, along with decreased synaptic puncta density, may underlie the cognitive impairments, motor deficits, and elevated seizure risk observed in GBM patients (53, 54).

Mechanistically, compression appears to reduce iN network activity by driving apoptosis and decreasing the number and size of synaptic puncta (55), though further work is needed to determine whether synaptic dysfunction precedes neuronal loss or vice versa. In some neurological diseases, decreased synaptic activity itself can contribute to neuronal loss (55). For instance, in Alzheimer’s Disease, the onset of synaptic dysfunction occurs prior to cortical neuron death, suggesting that reduction in synaptic activity can precipitate cell death (56).

On the molecular level, we detect robust activation of the HIF-1 signaling pathway in compressed neurons, with known pro- and anti-apoptotic effects in the context of neurologic diseases (57, 58). In some models, HIF-1α stabilization has been shown to promote recovery of spinal cord injury (59), cerebral ischemia (60–62), and TBI (63). However, other studies suggest that HIF-1α can directly contribute to secondary injury (58, 64). The hypoxia response signature observed in our data includes genes involved in anaerobic metabolism and angiogenesis, consistent with an adaptive role for HIF-1α. However, we also observe upregulation of proapoptotic genes, including *BNIP3* and *BNIP3L* (NIX)—both regulated by HIF-1α and known to induce mitophagy and cell death (65, 66). This aligns with our previous observation of increased LC3-positive vesicles in compressed mouse brains (4), suggesting that compression may trigger neuronal death through HIF-1α/BNIP3-mediated mitophagy. Given the pleiotropic functions of HIF-1α, our bulk RNA-seq data cannot resolve whether individual neurons activate both pro- and anti-apoptotic pathways simultaneously, or if distinct subpopulations activate these programs independently. Future studies incorporating snRNA-seq may help address this question.

HIF-1 signaling pathway is classically associated with cellular responses to hypoxia, although oxygen-independent, including mechanosensitive, modes of HIF-1 activation have been demonstrated previously (67). In vivo, the direct effects of solid stress are confounded by reduced blood perfusion, making it difficult to disentangle oxygen-dependent from oxygen-independent mechanisms of HIF-1 activation. Our in vitro compression device overcomes this challenge through the use of oxygen-equilibrated agarose hydrogels and the transwell system, which allow for gas and nutrient exchange under compression. Oxygen-sensing probe measurements confirmed that compressed cultures do not experience hypoxia at biologically significant levels. Using this experimental system, we demonstrate that the HIF-1 signaling pathway is activated by mechanical compression even in the absence of hypoxia, indicating a mechanosensitive mode of HIF-1 activation.

In addition to its direct effects on neurons, we observe that compression activates the proinflammatory and mechanosensitive (68) AP-1 pathway in both murine glial cultures and human astrocyte and microglia cell lines. Compressed glia upregulate cytokines such as *Cxcl1*, *Cxcl2*, *Cxcl3*, *Il23a*, *Il1b*, and *TNFA* which are known to contribute to neuroinflammation and may exacerbate neuronal injury (69–71). Notably, while both astrocytes and microglia activate the immediate-early AP-1 transcription factors *FOS* and *FOSB*, they exhibit distinct temporal dynamics. Microglia respond rapidly—within hours—to compression by transiently upregulating transcripts for TNF-α and IL-1β, cytokines with known neurotoxic roles (33), whereas astrocyte reactivity appears to be more sustained. We also observe species-specific responses: mGlia show upregulation of hypoxia response pathway, whereas human astrocytes did not upregulate hypoxia-related genes or show nuclear translocation of HIF-1α protein. This discrepancy may reflect limitations inherent to immortalized human cell lines, which may not fully recapitulate the behavior of primary glia. Future investigations using primary or iPSC-derived astrocytes and microglia may provide additional relevant models of human glial responses.

Our mechanistic insights are further supported by in vivo and patient data. In in vivo compression models, we observe elevated neuronal HIF1A, reduced density of VGLUT1+ puncta, and astrocytic inflammation in cortical areas exposed to compression. Interestingly, we observe these effects in regions adjacent to, rather than directly beneath, the site of applied pressure. This indicates that both compressive and tensile forces—present in these adjacent areas—contribute to the observed cellular responses. In GBM patients, we estimate solid stress using MLS—a well-recognized radiographic marker of mass effect. Since MLS is strongly influenced by increased fluid pressure due to peritumoral edema, we derived a surrogate for solid stress by residualizing MLS on edema volume. However, this approach assumes that the part of the MLS not accounted for by edema directly represents solid stress, which may overlook other biomechanical and anatomical factors that contribute to mass effect. To address these limitations, future investigations could employ direct solid stress measurement techniques (27, 28) to more accurately dissect the effects of solid stress on neural cells in patients.

In this study, we use Ngn2-induced human neurons—purely excitatory neurons, as they produce robust action potentials and reliably form functional synapses (30). Nevertheless, we observe significant variability in how neurons derived from different iPSC lines respond to compression. For example, neurons obtained from neonatal iPSCs #3 do not exhibit a significant increase in apoptosis observed in the iPSCs #2 and iPSCs #1 neurons. This variability may stem from intrinsic genetic or epigenetic differences between donor iPSC lines. In TBI, for example, genome-wide association studies have shown that common genetic variations substantially contribute to interpatient variability in clinical outcomes (72). While our sample size is limited, these observations underscore the need for further investigation into the role of genetic variation in neuronal response to injury.

Our in vitro and in vivo compression systems offer versatile platforms that can be tuned to model mechanical stresses associated with diverse neurological conditions beyond primary brain tumors. TBI lesions, for example, involve both cell hypertrophy (due to cellular swelling) and hyperplasia (due to leukocyte accumulation) that can generate compressive forces (2, 73). Similarly, conditions such as tumefactive MS, hydrocephalus, cerebral edema, and expanding hematomas each generate distinct mechanical stress profiles, often observed on MRI, that may directly influence cellular behavior and disease progression. To our knowledge, the specific magnitudes of compressive stresses in these pathological states have not been systematically quantified. Future studies could employ established biomechanical measurement techniques (8, 27) to characterize disease-specific stress parameters. These empirical values could then inform calibration of our in vitro and in vivo compression systems, enabling controlled studies of brain mechanobiology across a broad spectrum of neurological disorders, potentially revealing conserved mechanosensitive pathways that contribute to neural tissue dysfunction.

In summary, our findings indicate that chronic compression triggers a cascade of direct and indirect signaling events that ultimately lead to neuronal death, damage, and disruption of neuronal network activity. These findings point to potential therapeutic opportunities. For instance, our previous work has shown that lithium can partially rescue neuron loss and restore function under similar conditions (4). Our iN-based in vitro compression model provides a useful platform for screening neuroprotective compounds. The mechanistic insights from this study offer a foundation for developing interventions that target neuronal dysfunction in GBM and may also be applicable to other conditions involving mechanical brain injury, such as TBI or tumefactive MS.

## Materials and Methods

### Human Induced Neurons (iN Cells) and Lentivirus Generation.

Induced pluripotent stem cells (iPSCs) from three different healthy wild-type donors were used for experiments: WB02 (Asian male, 25 to 29 y; obtained from Joseph Wu, MD, PhD at the Stanford Cardiovascular Institute funded by NHLBI BhiPSC-CVD 75N9202D00019) referred to iPSCs #1, UKKi027-A (African female, 25 to 29 y; obtained from Millipore Sigma) referred to iPSCs #2, and ATCC-DYS0100 (ACS-1019; from neonate male; obtained from ATCC) referred to iPSCs #3. All work with human cell lines was approved by the Institutional Review Board (IRB) at the University of Notre Dame (Protocol ID: 21-11-6904). All iPSC lines used in this study were obtained as deidentified samples from publicly available repositories or collaborators, and no identifiable donor information was accessible to the researchers. iNs were produced following our previously published standard protocol (30, 74). Briefly, iPS cells were grown on 6-well dishes coated with Matrigel (Corning) in Stem Flex supplemented with rock inhibitor (thiazovivin, Bio Vision, 1:5,000). Medium was changed daily. When iPSCs formed colonies and became confluent, accutase (Innovative Cell Technologies) was used to dissociate cells from the plate. 1 mL accutase was added to each of the wells. Cells were put in the incubator for around 5 min. Cells were collected with 1 mL of medium in a 15 mL conical tube and centrifuged for 5 min at 300 × g. The supernatant was removed, and the cell pellet was resuspended in 1 mL Stem Flex (Thermo Fisher). 10 μL of the resuspension was used to count cells using the BioRad automated cell counter. For each cell line, 120 k cells were plated on 6-well 0.4 µm PET translucent transwell inserts (cellQUART) that had been coated in Matrigel (Corning) for 24 h. Cells used for live imaging were lentivirally transduced with 110 μL FUW-rtTA, 110 μL TetO-Ngn2, 110 μL FUW-GCamp8m [modified after pGP-CMV-jGCaMP8m, which was a gift from GENIE Project (Addgene plasmid # 162372; http://n2t.net/addgene:162372; RRID:Addgene_162372) Janelia Research Campus (75)], and 110 μL FUW-NLS-GFP. Robust generation of excitatory neurons from iPSCs using TetO-Ngn2 is an established and widely used method (28). Cells used for RNA-seq were lentivirally transduced with 110 μL rtTA and 110 μL Ngn2. The mastermixes included counted cells, lentivirus, thiazovivin (1:5,000), and Stem Flex. 1.5 mL of mastermix was plated onto each transwell. On day 0, the culture medium was replaced with DMEM/F12 (Thermo Fisher) supplemented with 1× N2, 1× NEAA (Thermo Fisher) containing human BDNF (10 ng/mL, PeproTech), human NT-3 (10 ng/mL, PeproTech), and mouse Laminin-1 (0.2 µg/mL, Thermo Fisher). Doxycycline (2 µg/mL, Sigma) was added on day 0 to induce TetO gene expression and retained in the medium until the end of the experiment. On day 1, a 48 h puromycin selection (1 µg/mL, InvivoGen) period was started. On day 3, mouse glia cells were added in neurobasal-A (NB) medium supplemented with B27/Glutamax (Thermo Fisher) containing Ara-C (2 µM, Sigma). After day 3, 50% of the medium in each well was exchanged every 2 d. FBS (5%, Corning) was added to the culture medium on day 5 to support astrocyte viability, and human neurons were assayed after 2 mo (Fig. 1 *A* and *B*).

Lentiviruses were produced in HEK293T cells (ATCC, VA) by cotransfection with three helper plasmids (pRSV-REV, pMDLg/pRRE, and vesicular stomatitis virus G protein expression vector) with 12 µg of lentiviral vector DNA and 6 µg of each of the helper plasmid DNA per 75 cm^2^ culture area using calcium phosphate. 12 mL of lentivirus-containing supernatant was harvested 48 h after transfection, aliquoted, and stored at −80 °C (76).

### Astrocyte and Microglia Cell Culture.

Immortalized human astrocytes (IHA, Creative Bioarray) were seeded at 200,000 cells per insert in 1 mL of DMEM supplemented with 5% FBS (Gibco), and 1× Pen/Strep (Corning) (77). Human C20 microglia (34–36) were seeded at 20,000 cells in 0.4 μm pore size transwell cell culture insert (CellQART, 4.5 cm^2^) in 1 mL of DMEM/F12 medium supplemented with 1× N2 (Gibco), 1% FBS, and 1× Pen/Strep. Once adherent, the cells were compressed for a defined period of time. For live cell morphology analysis, the cells were washed twice with 1× PBS, stained with 0.8 µg/mL Calcein AM (BioLegend) diluted in 1 mL of 1× PBS for 30 min at 37 °C, and washed twice again with 1× PBS. Images were taken at 20× magnification using a fluorescent microscope (Leica DMi8) and analyzed in ImageJ Software. Microglia circularity was analyzed by converting images to binary, despeckling, thresholding to obtain masks of the soma region, and measuring circularity of individual cells. Soma lengths and widths were measured manually using the ImageJ measuring tool.

### In Vitro Compression.

To apply a defined amount of compressive stress, we used a previously described in vitro compression device (78, 79). Briefly, a 1% agarose cushion (pre-equilibrated overnight in the incubator) was placed on top of the cells in the transwell insert, and a 3D-printed PLA weight was placed on top to apply 0.14 kPa of compressive stress for 24 h. This level of solid stress is meant to simulate the solid stress that we previously measured in murine GBM models (4) and GBM patients (28). Control cells were covered with an agarose cushion only (0 kPa).

### Live/Dead Staining.

Cell viability was assessed using Hoechst 33342 (Invitrogen) and PI. Hoechst 33342 is a cell-permeant nuclear dye and was used for data normalization. PI is a membrane impermeant dye and stains apoptotic and/or necrotic cells whose cell membrane is disrupted. To allow for discrimination of neurons vs. glia, we overexpressed nuclear localized (nuclear localization sequence—NLS) GFP in neurons. After weights and cushions were carefully removed together with any remaining medium, 0.5 mL of working solution containing 3 µg/mL PI and 1:1,000 Hoechst 33342 diluted in the imaging buffer (4 mM CaCl_2_ and 8 mM KCl in 129 mM NaCl, 25 mM HEPES, 30 mM glucose, 1 mM MgCl2, 10 µM Glycine) were added to the transwell insert, and 1 mL was added underneath the insert into the cell culture well. The cells were allowed to incubate for 10 min at 37 °C, 5% CO^2^ before imaging.

Image analysis was conducted by generating binary masks for Hoechst+, GFP+, and PI+ images using consistent threshold values across each batch of iNs. Thresholding was performed in *ImageJ* (version 1.54 g). All subsequent analyses were carried out using a custom Python script. The number of PI+ cells in each image was quantified to calculate apoptotic cell counts. The apoptotic cell fraction was determined as the proportion of Hoechst+ cells overlapping with PI+ cells in each image. The total iN cell counts corresponded to the number of GFP+ cells per image. Finally, the apoptotic iN fraction was defined as the proportion of GFP+ cells overlapping with PI+ cells per image.

### Calcium Imaging.

Following compression, the cells were used for calcium imaging. The weight was carefully removed from the compressed neurons. Existing media was aspirated from inside and outside of the transwell. Calcium imaging was performed as previously described (80, 81) and modified for agarose-covered transwell condition. Briefly, after lentivirally mediated expression of GCaMP8m for 2 mo, 1 mL of calcium imaging buffer (4 mM CaCl_2_ and 8 mM KCl in 129 mM NaCl, 25 mM HEPES, 30 mM glucose, 1 mM MgCl2, 10 µM Glycine) was pipetted outside the transwell, and 200 μL on to the agarose cushion in the transwell. Neurons were placed in the incubator for 3 to 5 min and then imaged in the transwells with an inverted epifluorescence microscope (Nikon EclipseTS2R) with a 488 nm filter at room temperature. GCaMP8m fluorescence was recorded for 2 min at a frame rate of 50 ms using NIS Elements 5.30.05. The ROI was set to 1,192 × 1,192 Mono 16-bit, the objective was 10×. For each condition, 7 fields of view were imaged for each biological batch and condition. All images were acquired using the same exposure time. Time-lapse imaging was performed in areas containing confluent neuron populations with few overlapping cell somata. This procedure was used for compressed and uncompressed samples.

Calcium imaging files were analyzed using MATLAB through custom-made code. Neurons were selected and then each were analyzed to generate statistics summary: frequency and mean/standard deviation of amplitudes for each neuron and synchronous spikes (dependent on the sigma threshold we set, 1.5). For each replicate, cell line, and compressed/uncompressed, the data were combined and graphed using GraphPad Prism: Amplitude, Frequency, Synchronous Amplitude, and Synchronous Frequency.

### Confocal Imaging and Immunocytochemistry (ICC).

After 60 d in culture, Ngn2 + rtTA induced neurons were collected for ICC and confocal imaging. The agarose cushions and weights were carefully removed from the transwells. The neurons were fixed in 4% paraformaldehyde (PFA) and 4% sucrose in PBS for 20 min at room temperature, washed three times with PBS. Cells were blocked in PBS containing 5% goat serum for 1 h at room temperature and stored in the blocking buffer for up to 2 wk. Primary antibodies were applied overnight at 4 °C, cells were washed in PBS three times, and fluorescent-labeled secondary antibodies (Alexa 488 or 647, 1:1,000) were applied for 2 h at room temperature in the dark. The following antibodies were used in immunocytochemistry experiments: MAP2 (CPCA-MAP2, EnCor. 1:5,000), synapsin (7739; 1:2,000). The transwells were carefully washed 3× with PBS and 1× with ultrapure water. The membranes of the transwells were carefully removed and cut into a square. The membrane containing neurons was mounted onto a coverslide with DAPI fluoromount medium and a square coverslip. Slides were left to dry for 24 h.

Confocal imaging was performed using a Nikon Ti2 eclipse A1RSi confocal microscope using NIS elements version 5.30.06. 64-bit. 1,024 × 1,024 images were taken with 60× objective with 0.8 µm step size and 7 to 10 steps per image, exported as .nd2 files and uploaded to NIS elements version 5.42.04 64-bit. Each image was analyzed by making a new document from the current view with Maximum Intensity Projection (MaxIP). The GFP threshold was defined as 1,000 to 4,095, Clean 0.11 μm, Separate 0.11 μm. Individual regions of interest (ROIs) were drawn around dendrites (10 ROIs per image). Binary ROI data were collected with Automated Measurement Results and exported to Microsoft Excel. To measure the length of the ROIs in µm, we used the polyline tool in Annotations and Measurements. Puncta density was calculated by dividing the Number of Objects by the length of ROI. Puncta area was calculated by dividing the Binary Area by the Number of Objects. Puncta intensity was calculated by dividing the Sum Intensity by the Number of Objects.

Images showing the morphology of human neurons (after Map2 and DAPI staining) were analyzed using MetaMorph Offline (Version 7.10.4.407) with the Neurite Outgrowth app. The parameters for dendrite characterization were as follows: Cell Body: Approximate maximum width: 40 μm, Intensity above local background: 150 gray levels, Minimum area: 20 μm^2^. Outgrowths: Maximum width: 10 μm, Intensity above local background: 150 gray levels. Minimum cell growth to log as significant: 10 μm. For each image, the total number of outgrowths, processes, process length, process branches, process straightness, and cell body area was calculated. The number of cells per image was manually counted using the ImageJ plugin Cell Counter.

Confocal immunohistochemistry (IHC) images were analyzed using ImageJ (version 1.54 g). Background signal was removed from all channels using the “Remove Background” function (radius = 50). GFAP+ % area was calculated as the GFAP+ signal area divided by the total image area. NeuN+ cell frequency was determined by dividing the number of NeuN+ cells by the total number of DAPI+ nuclei. For nuclear HIF1A quantification, neuronal nuclear masks were generated from NeuN+DAPI+ nuclei, and mean fluorescence intensity (MFI) was measured per image. VGLUT1 expression was quantified as image-wide MFI, excluding empty regions without tissue. Astrocyte segmentation in confocal IHC images was performed using a custom Python script with scikit-image 0.25.2 tools. Briefly, nuclei were identified using a fixed threshold in max intensity projections, and the mean intensity of the projected GFAP (including signal from optical sections above and below the nucleus) was computed for each nucleus. A fixed threshold was applied to determine whether each nucleus belonged to an astrocyte. For astrocyte nuclei, the mean intensity of the FOSB staining was then calculated and normalized to the FOSB intensity of all nuclei in the image.

### Hypoxia Evaluation.

Prior to compression, cells were cultured in transwell inserts and incubated overnight with 100 nM HypoxiTRAK™ (Novus Biologicals) to allow dye uptake. The following day, cells were subjected to mechanical compression by placing an agarose gel and a weight into the insert for 24 h. After the weight was removed, the cells were gently washed with phosphate-buffered saline (PBS) and detached using Trypsin-EDTA (0.25%). Following detachment, cells were washed with PBS. Zombie Aqua™ viability dye (BioLegend) was diluted 1:400 in PBS, and cells were resuspended in 50 µL of the diluted dye. Samples were incubated at 4 °C in the dark for 15 to 30 min, then washed once with 1% bovine serum albumin (BSA, VWR) in PBS. Cells were subsequently fixed in 100 µL of 4% PFA for 10 min at room temperature, washed again, and immediately analyzed by flow cytometry (Cytek Aurora).

### PCR.

Following compression, cells were lysed in 350 μL of TRI-reagent (Zymo Research, R2050-1-200) and the RNA was purified using an RNA isolation kit (Zymo Research, R2051). Gene expression was analyzed using TaqMan primers for Fosl2 (Hs01050117_m1), Fosl1, (Hs00759776_s1), Tnf, (Hs00174128_m1), Gapdh (Hs02786624_g1), Fos (Hs04194186_s1), Col4a1 (Hs00266237_m1), Il1b (Hs00266237_m1), Fosb (Hs00171851_m1). The raw PCR data were analyzed using the qPCR Design and Analysis app (Thermo Fisher Scientific). Individual measurements that were clear outliers (e.g., due to pipetting errors) were removed.

### Bulk RNA-seq.

RNA-seq library preparation and sequencing was performed at the Notre Dame Genomics and Bioinformatics Core Facility. Quality of RNA samples was confirmed using High Sensitivity RNA ScreenTape (Agilent) before Illumina libraries were prepared from 200 ng of RNA using the NEBNext Ultra II Directional RNA Library Prep kit with Sample Purification Beads (E7765S) and using the NEBNext Poly(A) mRNA Magnetic Isolation Module (E7490S). Libraries were confirmed using the Fragment Analyzer and quantified by qPCR prior to pooling and sequencing on an Illumina NextSeq500 using 50+50 paired end reads. On average, 1 NextSeq P4 XLEAP flow cell generates 1.5-1.8 Billion clusters/reads, for an average of 125 Million raw reads per sample across both lanes.

### Analysis of iN RNA-seq Data.

To deconvolve neuronal and glial transcripts, sequencing reads were binned by mapping to human (GRCh38.p14) and mouse (GRCm39) reference genomes using BBMap (v. 39.08). Reads that mapped to both references were discarded. Binned reads were aligned to respective reference genomes and counted using STAR (v. 2.7.2). For PCA, gene counts were downsampled to the same library size and batch effects were regressed out using removeBatchEffects function in limma (v. 3.62.2) R package. The Bioconductor R package DESeq2 (v. 1.46.0) was used to normalize count data and perform differential expression analysis. Gene set enrichment and overrepresentation analysis was performed using clusterProfiler R package. RRHO analyses were performed using the RRHO2 R package (v 1.0).

To infer the relative abundances of murine cell types in the mouse glial RNA-seq data, we applied nonnegative least squares (NNLS) regression using aggregate expression of the top 100 marker genes for each cell population from the Tabula Muris scRNA-seq dataset. First, mouse brain scRNA-seq data were normalized and clustered using SNN-based clustering in Seurat (v. 5.2.1). Marker genes for each cell population were identified through the Wilcoxon Rank Sum test. Pseudobulk aggregate expression profiles for 100 marker genes based on avg_log2FC were normalized, and relative abundances of the cell populations in the bulk RNA-seq data were inferred using NNLS, implemented via the lsei R package (v. 1.3-0). Motif enrichment analysis was performed using HOMER (v5.1) with the findMotifs module, scanning regions from 400 bp upstream to 100 bp downstream of the TSS. Motif lengths of 8 and 10 base pairs were used for the analysis.

### Analysis of GBM-CARE snRNA-seq Data.

Single-cell RNA-seq data were processed using Seurat (v5.3.1) (82). Cell type annotations were borrowed from the parent publications (50, 51), and data were normalized using Seurat’s default NormalizeData() function. Synaptic gene sets were obtained from GO annotations using the GO.db and org.Hs.eg.db packages, specifically targeting child terms of GO:0045202 (synapse). Cellular responses to stress gene sets were obtained in a similar manner by targeting child terms of GO:0033554 (cellular response to stress). The “solid stress” signature was derived from the top 50 upregulated genes (*P*adj < 0.05 for iN#1 and *P*adj < 0.1 for iN #2, baseMean > 25, sorted by log2FoldChange) identified through differential expression analysis of compressed iN #1 and iN #2 cells. Gene sets containing fewer than 10 genes were excluded from downstream analysis. Module scores were calculated using Seurat’s AddModuleScore() function for each gene set within individual samples, with analysis restricted to excitatory neurons in samples containing at least 10 cells. Spearman correlation matrix was computed for a set of all gene sets, and spearman correlation values were averaged across all samples.

### Analysis of Ivy GAP RNA-seq Data.

Anonymized BAM files were downloaded from the Allen Brain Atlas API and were counted using featureCounts (v2.0.1) and GRCh37.p5 reference genome. DESeq2 (v. 1.46.0) was used to normalize count data and perform differential expression analysis. Neuronal gene expression deconvolution was performed using BayesPrism (v. 2.2.2) and scRNA-seq ref. 83. To preprocess scRNA-seq data, the count matrix was log-normalized, scaled, and clustered using the shared nearest neighbor (SNN)-based algorithm in FindClusters Seurat (5.1.0) function. The clusters were annotated based on marker genes provided in the parent publication (83). For gene expression deconvolution, genes located on the sex chromosomes were excluded from the reference to avoid sex-specific transcriptional states, and noncoding, ribosomal protein-coding as well as mitochondrial genes were removed to minimize batch effects. Inferred gene expression values were rounded to the nearest integer and normalized using the DESeq2 workflow.

### Analysis of Ivy GAP MRI Data.

MLS was measured using a previously described method (84). MLS was defined as the maximum deviation of the septum pellucidum from the midline. MLS was calculated by drawing a line between the anterior and posterior falx cerebri, then measuring the perpendicular distance to the point of maximal deviation from the septum pellucidum. To separate the effects of fluid pressure (edema) from solid stress (e.g., mass effect from solid components of the tumor), we regressed MLS on edema volume and used the residuals as a proxy for solid stress. Patients with positive residuals—indicating more shift than expected from edema alone—were classified as “high” solid stress; those with zero or negative residuals were classified as “low.” To study gene expression at the tumor margin in these two groups, we analyzed RNA-seq and ISH data from histologically annotated “leading edge” regions, where the malignant-to-normal cell ratio is approximately 1 to 2 per 100 (85).

### Mice.

Animal experiments conducted in this study were in accordance with National Institute of Health Guidelines for the Care and Use of Laboratory Mice and approved by the Institutional Animal Care and Use Committee (IACUC) at the University of Notre Dame. Mouse glial cells were cultured from the forebrain of newborn wild-type CD1 mice. Briefly, newborn mouse forebrain homogenates were digested with papain and EDTA for 15 min, cells were dissociated by harsh trituration to avoid growing of neurons, and plated onto T75 flasks in DMEM supplemented with 10% FBS. Upon reaching confluence, glial cells were trypsinized and replated at lower density once to remove potential trace amounts of mouse neurons before the glia cell cultures were used for coculture experiment with human neurons.

### In Vivo Compression Model.

Cranial compression windows were surgically implanted in adult C57BL/6 mice, as previously described (4, 9). Compression was applied daily for 3 wk by tightening the screw 67 degrees per day, resulting in an estimated volumetric displacement of approximately 1.3 mm^3^/d. At the study endpoint, mice were euthanized using carbon dioxide, followed by bilateral pneumothorax (per IACUC protocol #24-06-8649), and the entire head was fixed in PFA at 4 °C for 48 h. Brains were then dissected, rinsed in PBS, and stored until further processing. Fixed tissues were processed using standard histological protocols and embedded in paraffin.

### Tissue Immunohistochemistry.

Paraffin-embedded tissue sections (10 µm) were deparaffinized in xylene and rehydrated through a graded ethanol series (100%, 95%, 70%, 50%, and 0%). Antigen retrieval was performed by boiling the slides in citrate buffer (10 mM sodium citrate, 0.05% Tween 20, pH 6.0) for 20 min, followed by gradual cooling to room temperature. Slides were then washed three times with PBS, permeabilized with 0.5% Triton X-100 (Thermo Fisher Scientific, 0694) for 20 min, and washed again three times with PBS. Blocking was performed using 10% normal goat serum (Sigma-Aldrich, G6767), 0.2% Triton X-100 in PBS for 1 h at room temperature. Primary antibody staining was carried out overnight at 4 °C using the following antibodies diluted in PBS: rabbit anti-HIF1A (1:100, Invitrogen, PA1-16601), rat anti-NeuN (1:500, Abcam, ab279297), chicken anti-GFAP (1:200, EnCor Bio, CPCA-GFAP), rabbit anti-FOSB (1:200, Abcam, ab184938), and guinea pig anti-VGLUT1 (1:500, Millipore Sigma, AB5905). For each tissue block, a no-primary antibody control was included to assess nonspecific staining. The next day, slides were washed three times with PBS (5 min each) and incubated for 1 h at room temperature with secondary antibodies diluted in PBS: goat anti-rat AlexaFluor 488 (1:500, Abcam, ab150157), goat anti-rabbit AlexaFluor 647 (1:500, Abcam, ab150079), goat anti-chicken IgY AlexaFluor 594 (1:500, Invitrogen, A-11042), and anti-guinea pig AlexaFluor 546 (1:500, Invitrogen, A-11073). Following secondary antibody incubation, slides were washed three times with PBS, stained with DAPI (1:1,000), and mounted using Fluoromount-G™ Mounting Medium (Invitrogen). Slides were allowed to cure overnight, and images were acquired using Leica Stellaris 8 DIVE and Nikon Ti2 confocal microscopes.

### Statistical Analysis.

Unless otherwise specified, the data are presented as the mean ± SEM. Student’s two-tailed *t*tests and Mann–Whitney *U* tests were used to assess the significance. *P* < 0.05 was considered significant. Statistical analyses were performed using Graphpad Prism or R programming language (v 4.4.3).

## Supplementary Material

Appendix 01 (PDF)

Dataset S01 (XLSX)

Movie S1. Live calcium imaging of uncompressed iN #1 and compressed iN #1. Both control and compressed iNs were grown in culture for 60 days. On Day 59, iNs were compressed for 24 hours and then imaged along with the uncompressed iNs. iNs were imaged in a calcium imaging buffer for 2 minutes with a 50 msec framerate. Video is displayed as a heatmap of fluorescent intensity where warmer colors represent higher intensities. Scale bar = 100 μmm.

## Data Availability

All transcriptomic data have been deposited in the Gene Expression Omnibus with accession numbers GSE313799 (86) and GSE313613 (87). Differential expression statistics are available in Dataset S1. All other raw data are available in the GitHub repository at https://github.com/plezar/Neuron-Compression-PNAS (88).
