# Supplementary material for: Mechanical compression induces neuronal apoptosis, reduces synaptic activity, and promotes glial neuroinflammation in mice and humans
Source: Proc Natl Acad Sci U S A. 2026 Jan 2;123(1):e2513172122. doi: 10.1073/pnas.2513172122 (PMC12773780; doi:10.1073/pnas.2513172122)
Supplement: Supplementary file 1 — Appendix 01 (PDF) [file pnas.2513172122.sapp.pdf]

Supporting Information for

Mechanical compression induces neuronal apoptosis, reduces synaptic activity, and promotes glial neuroinflammation in mice and humans

Maksym Zarodniuk[1*], Anna Wenninger[2*], Julian Najera[1], Jihaeng Lee[2], Jack Markillie[3], Cameron MacKenzie[2], Jenny Bergqvist-Patzke[2], Bianca Batista[4], Megna Panchbhavi[5], R'nld Rumbach[1], Alice Burchett[1], Charles Sander[2], Meenal Datta[1#], Christopher Patzke[2#]

[1]Department of Aerospace and Mechanical Engineering, [2]Department of Biological Sciences, [3]Department of Chemistry and Biochemistry, [4]Department of Chemical and Biomolecular Engineering, [5]Department of Applied and Computational Mathematics and Statistics, University of Notre Dame

* These authors contributed equally to this work
[#]Correspondence to:
Christopher Patzke, 109A Galvin Life Science Center, University of Notre Dame, Notre Dame, IN 46556
Email: cpatzke@nd.edu
Meenal Datta, 2027E McCourtney Hall East, University of Notre Dame, Notre Dame, IN 46556
Email: mdatta@nd.edu

This PDF file includes:

Figures S1 to S5
Legends for Movie S1
Legends for Dataset S1

Other supporting materials for this manuscript include the following:

Movie S1
Dataset S1

Figures

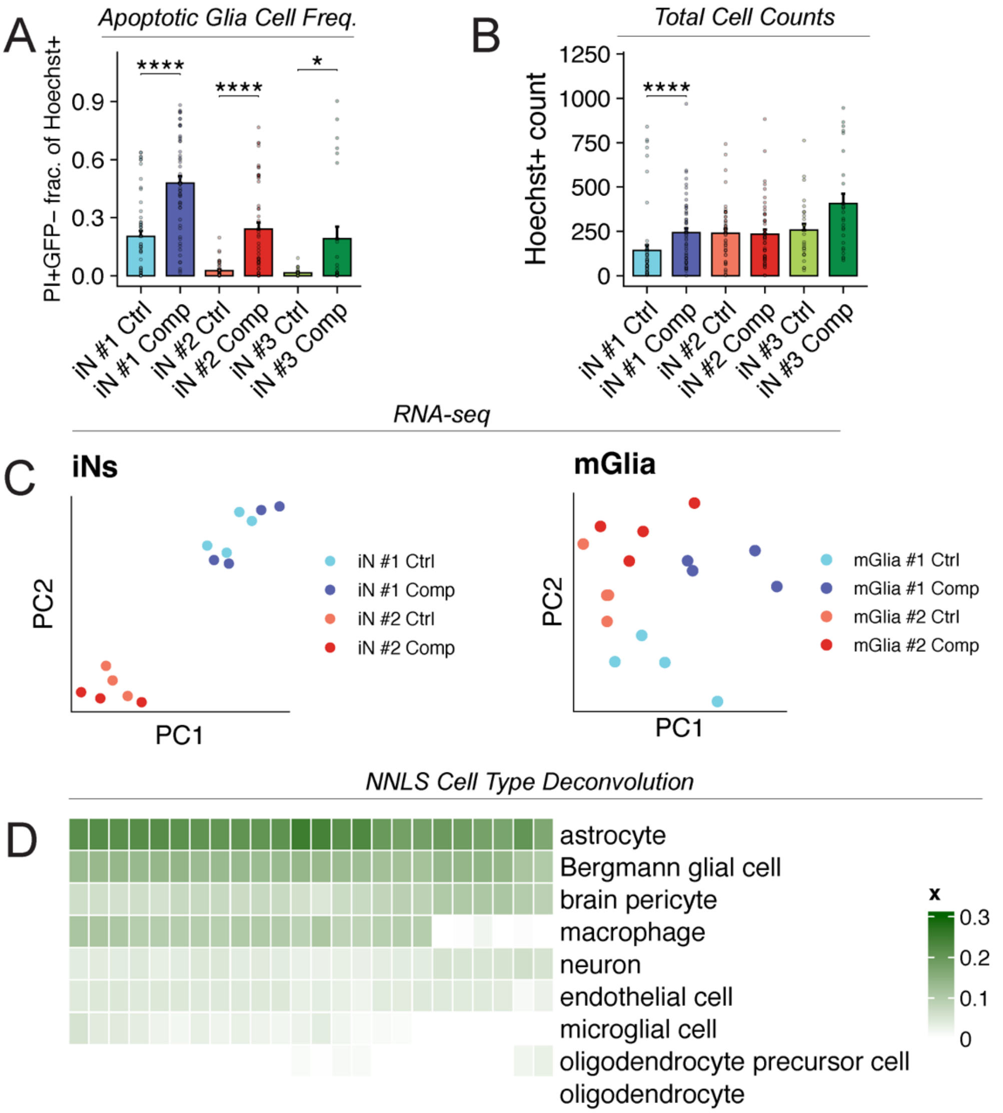

Supporting Figure 1. Quantification of glial cell viability and molecular characterization of neuronal and glial populations. (A) Fraction of apoptotic glial cells is quantified as the proportion of Hoechst+ cells overlapping with PI (PI+) but not GFP (GFP-) per field of view. **(B)** Total cell numbers are quantified as the number of Hoechst+ cells per field of view. **(C)** Principal component analysis of human neurons (iN) and murine glia (mGlia) shows that iN transcriptomes separate based on source iPSC donor, while murine glia separate based on experimental condition. **(D)** Non-negative least squares (NNLS)-based cell type deconvolution of bulk RNA-seq data from murine glial cultures reveals strong enrichment of the astrocyte gene expression signature. Mann–Whitney U test with Holm–Bonferroni adjustment for multiple comparisons using N = 4 biological replicates for iNs #3, n=7 biological replicates in iNs #2 and iNs #1 neurons per group; the data are based on 7 independent experiments; Error bars: mean ± SEM. Statistical significance is indicated as follows: * = P < 0.05; *** = P < 0.001.

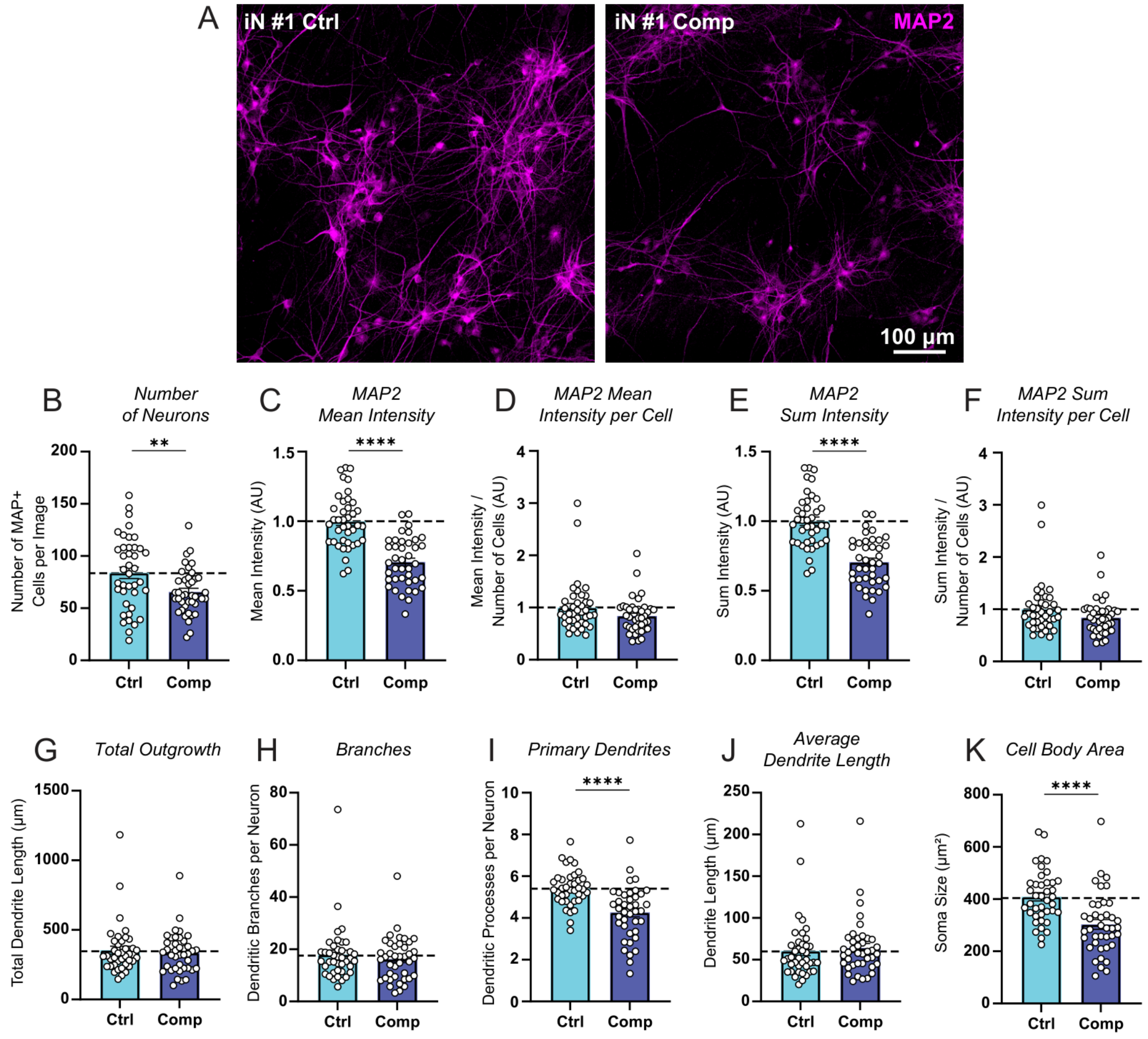

Supporting Figure 2. Morphological analysis of compressed iNs shows reduced dendritic arborization. (A) Representative confocal images of iN #1 uncompressed and compressed human neurons. (Magenta = MAP2) (Scale bar = 100 µm). **(B-F) (B)** Quantification of the number of neurons, **(C)** MAP2 mean intensity, **(D)** MAP2 mean intensity per cell, and **(E)** MAP2 Sum Intensity, and **(F)** MAP2 Sum Intensity per cell. **(G-K)** Morphology of uncompressed and compressed human neurons were analyzed using MetaMorph software. **(G)** No difference in the total outgrowth between conditions. **(H)** Statistical analysis reveals no difference in the number of dendritic branches, **(I)** but a decrease in the number of primary dendrites in the compressed iNs. **(J)** The length of each dendrite does not change, **(K)** however the total cell body area decreases significantly in the compressed condition. Data shown are presented as individual values (each representing one image for confocal analyses) with indicated mean ± SEM. Student's unpaired t test n = 40 uncompressed images, n = 40 compressed images, from 2 biological replicates for each condition. ns = $P \geq 0.05$; * = $P < 0.05$; ** = $P < 0.01$; *** = $P < 0.001$; **** = $P < 0.0001$.

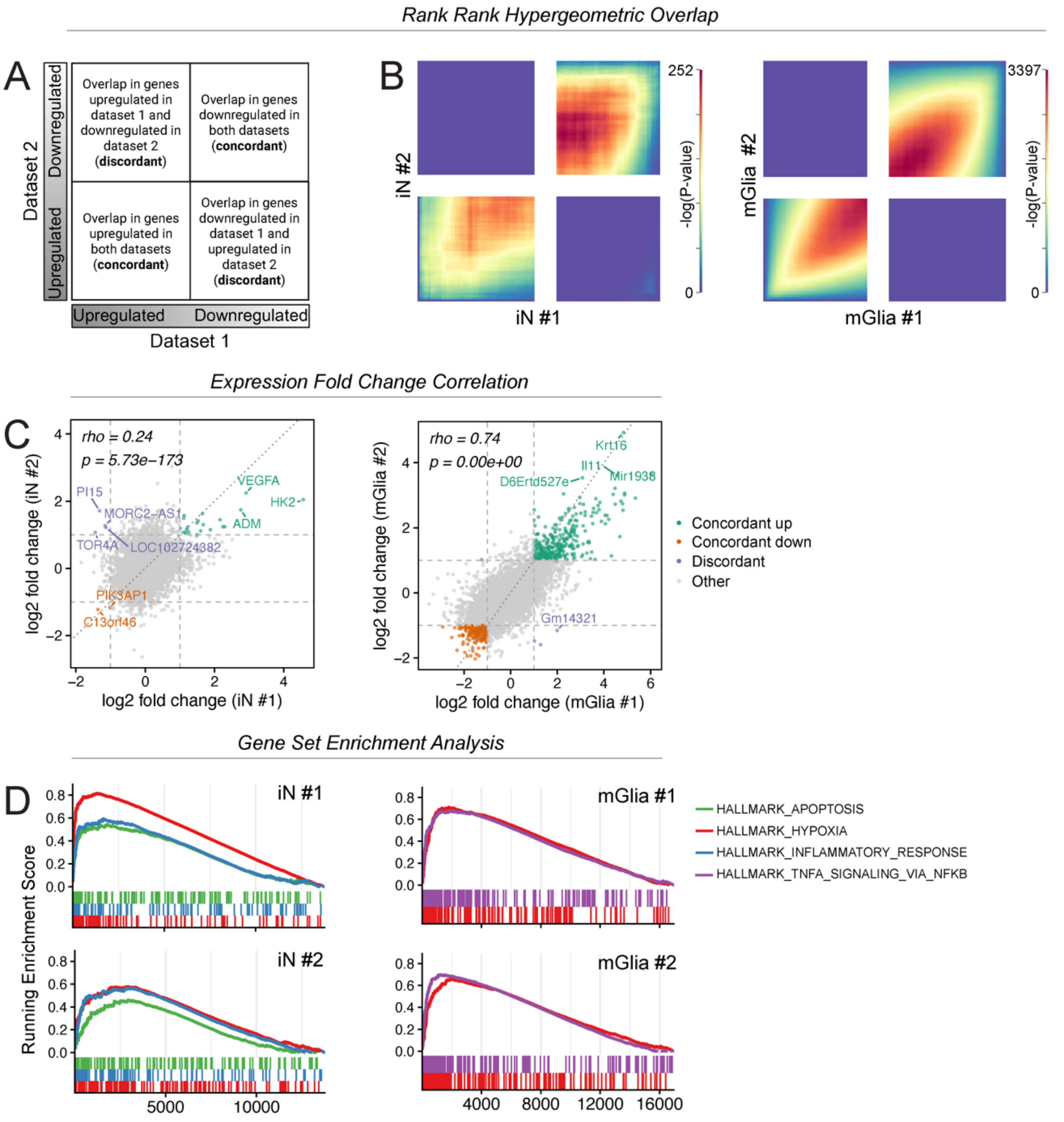

Supporting Figure 3. Cross-donor reproducibility of neuronal and glial transcriptional responses to compression. (A) Schematic of rank–rank hypergeometric overlap (RRHO) analysis. Each gene is ranked by expression fold change (log_2FC), and overlap between two ranked gene lists is quantified using a hypergeometric test at all possible rank thresholds. Enrichment in the upper-right and lower-left quadrants reflects concordant up- and downregulation, respectively. **(B)** RRHO2 heatmaps showing concordance across independent iPSC donors. Left: human induced neurons (iN #1 vs iN #2). Right: murine glia (mGlia #1 vs mGlia #2). Axes correspond to gene ranks from each dataset; color scale indicates $-\log_{10}$ *p* values of overlap significance. Strong enrichment along the diagonal indicates highly concordant gene regulation across donors. **(C)** Correlation of differential expression magnitudes between donor pairs. Each point represents a gene, colored by directionality: concordant upregulated (green), concordant downregulated (orange), discordant (purple), or other (gray). Dashed lines denote $log_2FC=\pm1$ thresholds. Spearman's rho and *p*-values quantify agreement in expression fold changes across

donors. **(D)** Gene set enrichment analysis (GSEA) of conserved solid stress-responsive pathways in both neurons (iN #1, iN #2) and glia (mGlia #1, mGlia #2).

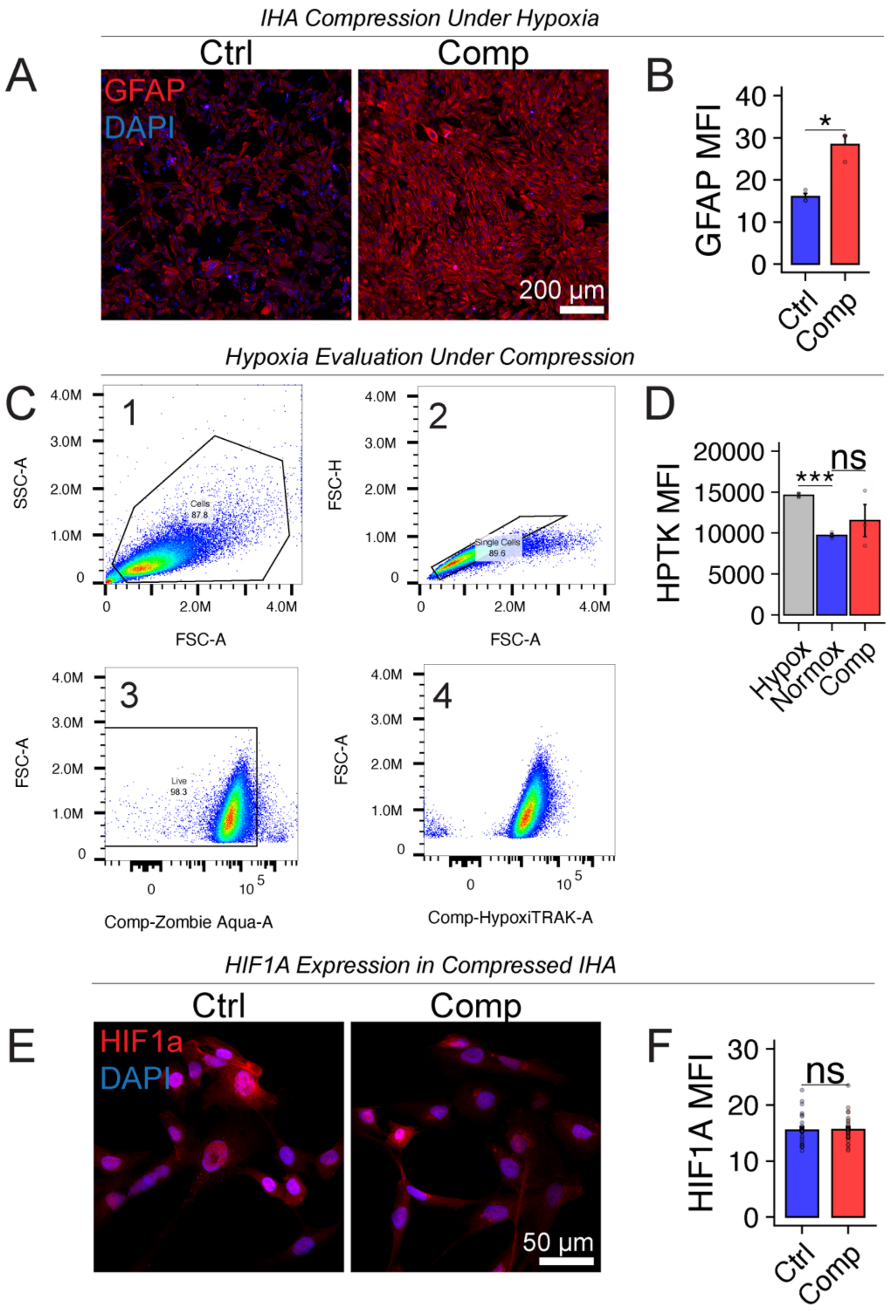

Supporting Figure 4. Astrocyte activation in response to compression occurs independently of hypoxia. (A–B) Immunofluorescence staining of GFAP in immortalized human astrocytes (IHA) cultured under hypoxia (1% O_2), with or without compression (n = 3 biological replicates per group). **(C–D)** Flow cytometry gating strategy and quantification of HypoxiTRAK fluorescence in IHA cultured under normoxia (21% O_2, n=3), hypoxia (1% O_2, n=2), or compression (n=3). Compression does not induce biologically significant hypoxia. **(E–F)** Immunofluorescence analysis of HIF1A shows no increase in nuclear HIF1A accumulation in compressed astrocytes. Student's two-tailed t-test; error bars: mean ± SEM. Statistical significance is indicated as follows: * = $P < 0.05$, *** = $P < 0.001$, ns = $P \geq 0.05$.

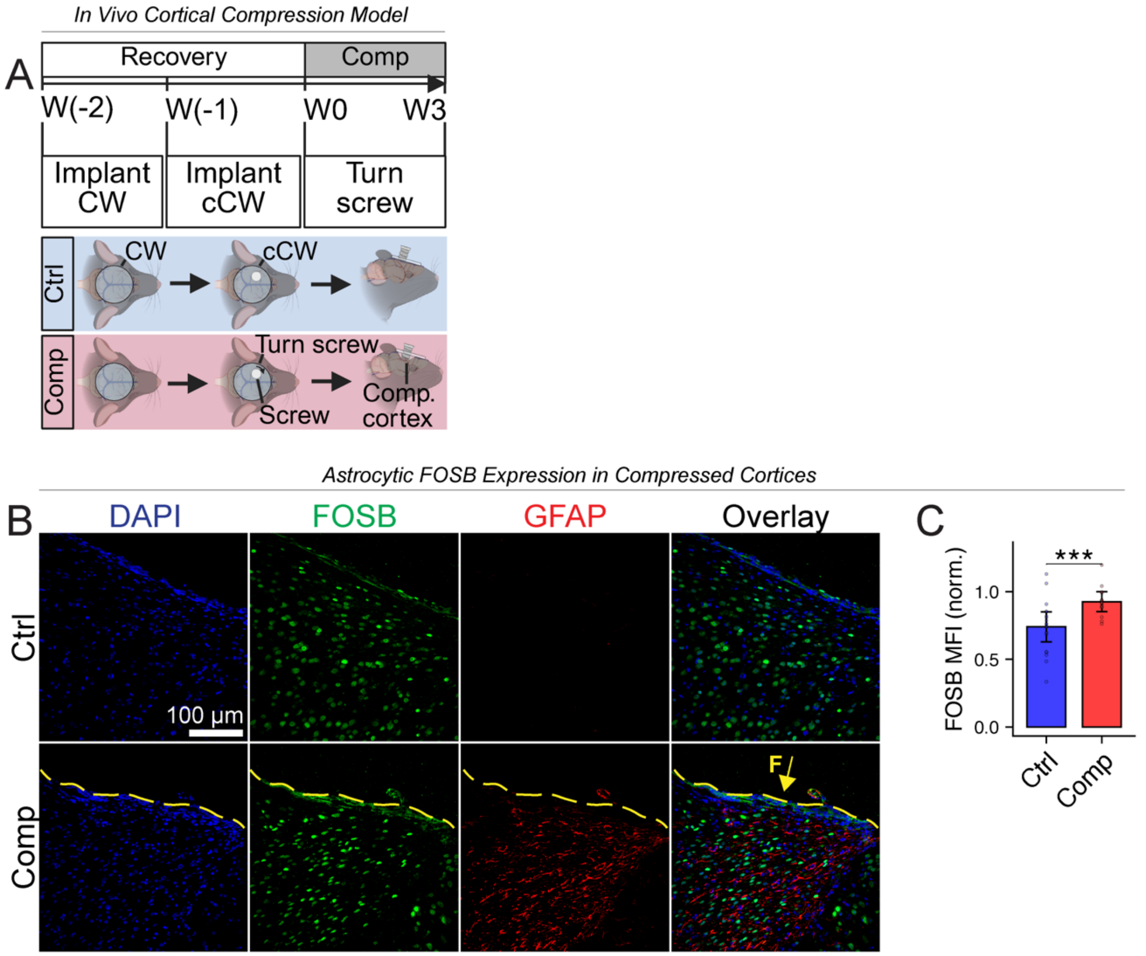

Supporting Figure 5. *In vivo* cortical compression leads to FOSB activation in astrocytes. (A) Schematic and timeline of the *in vivo* cortical compression system. The compression cranial window (cCW) device consists of a set screw mounted on a cranial window (CW), separated from the brain surface by a biocompatible, deformable membrane. The screw gradually expands the compression volume at approximately 1.3 mm^3/day over 3 weeks, mimicking the growth rate of nodular intracranial GBM models (4). Control mice receive the cCW implant without deployment of the compressive screw. Mice recover for one week in between the initial CW surgery (W(-2)) and replacement with the cCW (W(-1)). **(B)** Representative images showing FOSB and GFAP expression in the compressed cortex (Scale bar: 100 µm). The area of cortical indentation (compression divot) is marked with a yellow dashed line, and the site of applied compression is indicated by a yellow arrow; F: compressive force. **(C)** Quantification of immunohistochemistry data shown in panel B. FOSB expression in astrocyte nuclei is quantified relative to total FOSB expression for each image (**Methods**). Statistical significance was determined by Mann–Whitney U test. *** = $P < 0.001$. norm.=normalized.

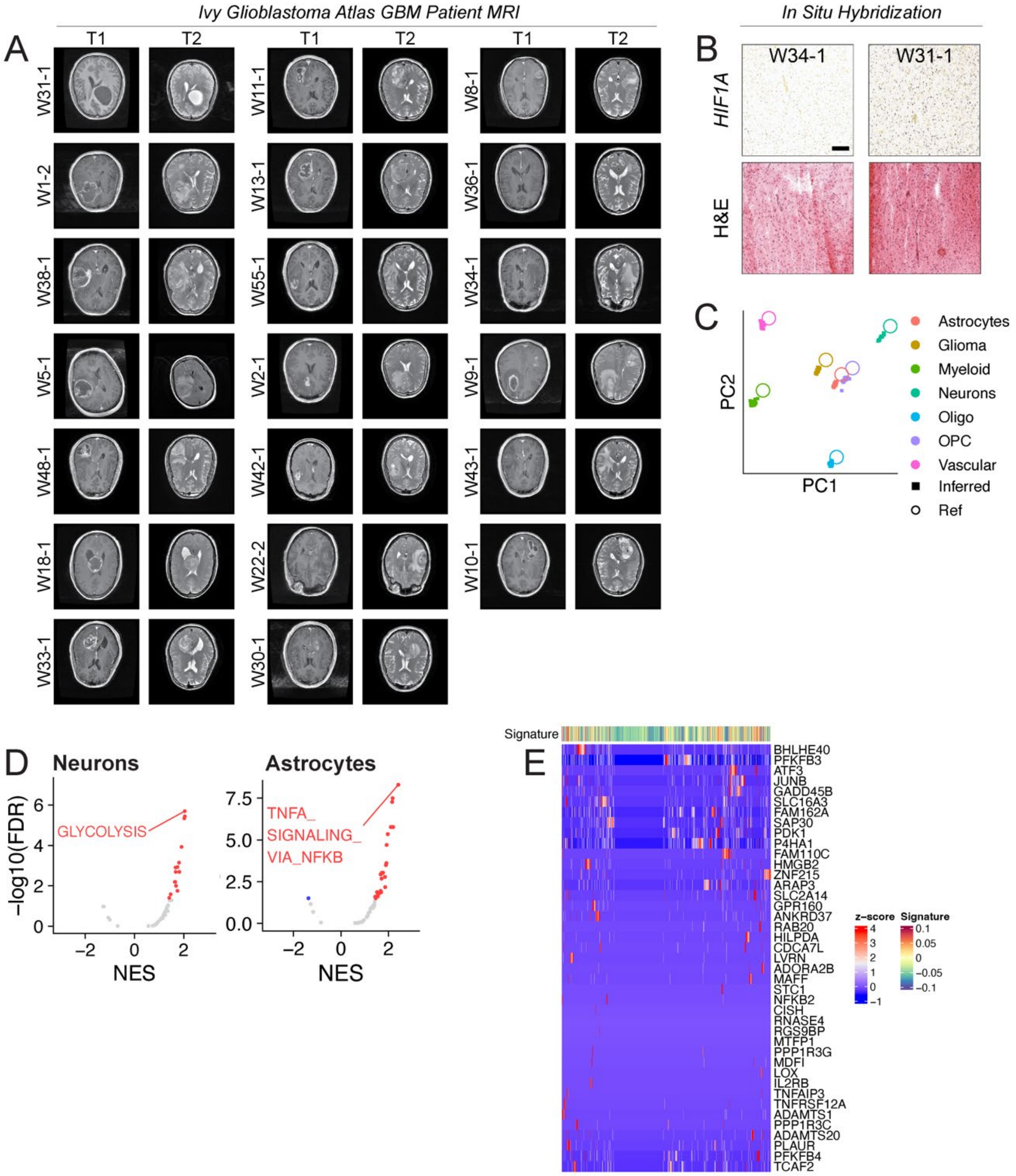

Supporting Figure 6. Solid stress estimation in the Ivy Glioblastoma Atlas Project. (A) Solid stress estimation in the Ivy Glioblastoma Atlas Project patient cohort. Patients are ranked by estimated solid stress levels (top-to-bottom, then left-to-right). T1-weighted and T2-weighted MRI images are shown for each patient to illustrate the contributions of tumor burden and peritumoral edema. “W##-#” represents a patient identifier; data from 20 patients are included. **(B)** Representative in situ hybridization images showing HIF1A expression in patients with low (left) and high (right) estimated solid stress. **(C)** Joint embedding of pseudobulk reference gene expression profiles (triangles) and deconvolved bulk gene expression profiles (squares) in the principal component shows that BayesPrism infers cell-type-specific gene expression in heterogeneous cell types. **(D)** GSEA of BayesPrism-deconvolved bulk RNA-seq data reveals activation of glycolysis and NF-κB signaling pathways in neurons and astrocytes in regions of high

solid stress. **(E)** Expression of solid stress response signature genes in snRNA-seq data of excitatory neurons. Solid stress response scores are shown as top heatmap annotation.

Movie S1 (separate file). Live calcium imaging of uncompressed iN #1 and compressed iN #1. Both control and compressed iNs were grown in culture for 60 days. On Day 59, iNs were compressed for 24 hours and then imaged along with the uncompressed iNs. iNs were imaged in a calcium imaging buffer for 2 minutes with a 50 msec framerate. Video is displayed as a heatmap of fluorescent intensity where warmer colors represent higher intensities. Scale bar = 100 μm.

Dataset S1 (separate file). Differential expression analysis statistics for iNs and glia (#1-#3). baseMean: the average of the normalized count values; log2FoldChange: the effect size estimate; lfcSE: the standard error estimate for the log2 fold change estimate; stat: the value of the Wald test statistic for the gene; pvalue: P-value of the test for the gene; padj: adjusted P-value for multiple testing for the gene; DE: binary factor indicating upregulation (1) or downregulation (-1) of a gene.
